# Thirteen Years of Reconstructive Breast Surgery in a Korean Tertiary Practice: Procedure Spectrum, Shifting Practice, and Modality Selection in 2216 Patients

**DOI:** 10.3390/medicina62101871

**Published:** 2026-09-27

**Authors:** Byeongju Kang, Woosung Jang, Jongmoo Park, In Hee Lee, Joon Suk Moon, Jeeyeon Lee, Ho Yong Park, Jeong Yeop Ryu, Kang Young Choi, Jung Dug Yang, Ho Yun Chung, Joon Seok Lee

**Affiliations:** 1 Department of Surgery, School of Medicine, Kyungpook National University, Kyungpook National University Chilgok Hospital, Daegu 41404, Republic of Korea; libertas033@knu.ac.kr (B.K.); joonsukm@knu.ac.kr (J.S.M.); j.lee@knu.ac.kr (J.L.); phy123@knu.ac.kr (H.Y.P.); 2 Department of Plastic and Reconstructive Surgery, School of Medicine, Kyungpook National University, Daegu 41566, Republic of Korea; jjh2us@knuh.kr (W.J.); prsryu@knu.ac.kr (J.Y.R.); kychoi@knu.ac.kr (K.Y.C.); lambyang@knu.ac.kr (J.D.Y.); hy-chung@knu.ac.kr (H.Y.C.); 3 Department of Radiation Oncology, School of Medicine, Kyungpook National University, Daegu 41566, Republic of Korea; fauny11@naver.com; 4 Department of Oncology/Hematology, School of Medicine, Kyungpook National University, Kyungpook National University Chilgok Hospital, Daegu 41566, Republic of Korea; ihleeoncology@knu.ac.kr

**Keywords:** breast reconstruction, oncoplastic surgery, chest wall reconstruction, latissimus dorsi flap, direct-to-implant, mortality, confounding by indication

## Abstract

*Background and Objectives:* Reconstructive breast surgery covers a far wider range of operations than the implant-versus-autologous dichotomy: oncoplastic techniques, several pedicled flap designs, and chest wall coverage of defects resisting primary closure are rarely reported together. We described the complete reconstructive spectrum of a single tertiary center over thirteen years and asked whether the reconstructive choice carries prognostic information. *Materials and Methods:* All reconstructive procedures performed by the breast reconstruction service of a Korean tertiary hospital between December 2012 and July 2026 were reviewed retrospectively. Free-text operative descriptions were mapped onto a 26-category classification, and each patient received a primary technique from her index operation, separated by reconstructive intent: restoration of breast form or coverage of a defect after wide resection. Mortality was ascertained from institutional records; termination of National Health Insurance eligibility was examined separately as a candidate surrogate, with the last institutional attendance retrieved for every patient so flagged. Associations with recorded mortality were assessed by logistic regression. *Results:* In total, 2216 patients underwent 2523 procedures. The index operation was autologous reconstruction in 907 patients (40.9%), oncoplastic breast-conserving surgery in 623 (28.1%), implant-based reconstruction in 564 (25.5%), and chest wall reconstruction in 14 (0.6%). Autologous reconstruction fell from 67 of 114 index procedures in 2014 to 15 of 160 in 2026, whereas implant-based reconstruction rose from four to 70. Recorded mortality was 1.8% overall and ranged from none after several oncoplastic techniques to 28.6% after chest wall reconstruction. Patients whose eligibility had lapsed resembled survivors rather than the deceased; all 41 had attended in person after reconstruction, at a median of 4.3 years. *Conclusions:* The reconstructive operation summarizes the oncological situation rather than determining survival independently. Chest wall reconstruction merits reporting apart from breast reconstruction, and lapsed insurance eligibility performed poorly as a surrogate for mortality.

## 1. Introduction

Breast cancer is the commonest malignancy in Korean women, is diagnosed most often in the fifth decade of life, and is increasingly detected at an early stage, with stage 0 or I disease accounting for roughly two-thirds of new diagnoses [1]. Post-mastectomy breast reconstruction has consequently become a standard component of breast cancer care in Korea. The National Health Insurance Service extended coverage to reconstruction in April 2015, and the national reconstruction rate subsequently rose from 19.4% to 53.4% within three years, and the case mix moved decisively toward implant-based techniques [2,3]. These national figures derive from administrative claims and remain necessarily coarse: they separate implant from autologous reconstruction, yet leave the actual operative repertoire invisible.

The gap matters because reconstruction represents far more than a binary choice. A patient undergoing breast-conserving surgery may receive glandular reshaping, a rotation flap, a lateral thoracodorsal flap, a lateral intercostal artery perforator flap, therapeutic reduction, or a tennis-racket incision, each carrying distinct indications and distinct esthetic consequences. A patient undergoing mastectomy may receive a direct-to-implant reconstruction, a two-stage expander reconstruction, one of several latissimus dorsi flap designs, an abdominal flap, or a combination of these. A small number of patients undergo an operation that falls outside breast reconstruction altogether: resection of locally advanced or recurrent disease can leave a defect too large for primary closure, and the reconstructive goal then becomes durable soft-tissue coverage of the chest wall rather than restoration of breast form.

Thirteen consecutive years of reconstructive practice at a single Korean tertiary center were therefore reviewed with three aims: to describe the full spectrum of techniques at the level at which surgeons actually perform them; to document how that spectrum has changed; and to test whether the reconstructive modality carries prognostic information once the oncological context is taken into account. A fourth question arose during the analysis. Our institutional records flagged a group of patients whose National Health Insurance eligibility had terminated, a status often assumed in Korean practice to indicate mortality, and the cohort itself was used to test that assumption.

## 2. Materials and Methods

### 2.1. Study Design and Cohort

This retrospective observational study drew on a prospectively maintained operative database of the breast reconstruction service at Kyungpook National University Chilgok Hospital, Daegu, Republic of Korea. All reconstructive procedures performed by the service between 22 December 2012 and 9 July 2026 were eligible. Male patients and procedures performed for gynecomastia were excluded, since the series concerns surgery of the female breast; three patients were excluded on this ground. The study was approved by the Institutional Review Board of Kyungpook National University Chilgok Hospital (IRB File No. KNUCH 2026-07-093; approved 31 July 2026), and the requirement for informed consent was waived owing to the retrospective design. The study was conducted in accordance with the Declaration of Helsinki, and is reported in accordance with the STROBE statement for observational studies; the completed checklist is provided as Supplementary Material. Subsets of this cohort have been reported previously in addressing different questions, namely the boomerang latissimus dorsi design [4], endoscopic and robotic latissimus dorsi harvest [5,6], and selection between the prepectoral and subpectoral planes [7]. A separate analysis of esthetic outcome after post-mastectomy radiotherapy, drawn from a subset of this cohort, is currently under peer review elsewhere. None of these reports described the reconstructive spectrum, the change in practice, or mortality, and no analysis presented here has appeared or been submitted elsewhere.

Three units of analysis are distinguished throughout, since a single patient may undergo several operations. Patients (n = 2216) were the unit for demographic characteristics and for mortality, and patient-level variables were taken from the index operation. The reconstruction episode (n = 2245) is the unit for technique attribution; a second episode was counted where a patient underwent a further reconstruction for metachronous contralateral disease or for recurrence, as distinct from a staged completion or a revision of an existing reconstruction. The individual operation (n = 2523) is the unit of measurement used for calculating surgical workload and reoperation counts. The figure of 2216 used throughout this paper covers oncoplastic breast-conserving surgery, chest wall coverage, and adjunctive procedures as well as mastectomy-based reconstruction, and spans the whole period from 2012 to 2026. Several operations combine more than one technique, such as a latissimus dorsi flap with an implant or an oncoplastic resection with contralateral balancing; a single operation may therefore contribute to more than one technique count. Breast volume was measured preoperatively as part of operative planning by the water-displacement method. A cast of the breast was formed with adhesive tape, removed intact, and filled with water; the volume of water required was read directly. This conventional technique remains, in our experience, the measurement corresponding most closely to the volume that must actually be replaced at operation. Measurements were available for 4092 breasts in 2075 of the 2216 patients (93.6%).

### 2.2. Procedure Classification

Operative descriptions were recorded as free text and showed substantial orthographic variation, including recurrent misspellings of “implant” and “reconstruction”. Text was normalized and mapped by a rule-based algorithm onto 26 mutually exclusive technique categories, which were then organized in two tiers. The upper tier records reconstructive intent and separates restorative procedures, whose object is the form of the breast, from coverage procedures, whose object is closure of a defect that resists primary closure. The lower tier records strategy within the restorative intent: implant-based breast reconstruction, autologous breast reconstruction, and oncoplastic breast-conserving surgery. Chest wall reconstruction constituted the sole coverage-intent category. A residual group held adjunctive procedures comprising nipple–areola reconstruction, fat grafting, contralateral balancing, and revision surgery. Chest wall reconstruction was defined as a procedure whose purpose was coverage of a soft-tissue defect that resisted primary closure after wide resection, rather than restoration of breast volume or contour; these operations were identified by explicit documentation in the operative record and were confirmed individually. An operation so recorded was assigned to coverage intent only where it followed a resection performed at that operation; where the same wording described work on an established prosthetic reconstruction, the operation was classified as revision and the patient retained the primary technique of her index reconstruction. Selection into this category is dictated by the extent of resection rather than by patient preference or by any comparison of reconstructive options. The same donor tissue may serve both intents, since a latissimus dorsi flap reconstructs a breast in one setting and covers a chest wall defect in another; the indication, the oncological setting, and the reconstructive goal nevertheless differ fundamentally, and these cases were therefore analyzed as a distinct category rather than pooled with breast reconstruction. This arrangement keeps operations that share a donor site yet serve opposite purposes separate throughout the analysis. For an operation containing elements of more than one category, a fixed clinical hierarchy assigned the reconstructive intent of highest complexity as the primary technique. The 26 categories, their definitions, and the text patterns by which operations were assigned to them are set out in full in Table S1. All mappings were reviewed against the source text; 18 of 2900 technique components (0.6%) resisted assignment and appear as “other”.

### 2.3. Surgical Techniques

#### 2.3.1. Techniques of Implant-Based Reconstruction

Immediate single-stage reconstruction placed a silicone implant at the mastectomy site, either prepectorally or within a subpectoral pocket; skin-sparing approaches that permit such immediate reconstruction carry local recurrence rates comparable to conventional mastectomy [8]. The prepectoral neo-pocket positioned the implant entirely superficial to the pectoralis major, with an acellular dermal matrix (ADM) providing anterior coverage and inferolateral support. Subpectoral placement followed one of two configurations, the relative merits of which have been examined against prepectoral placement in two-stage prosthetic reconstruction [9]; criteria for selecting between the two planes have also been proposed for Korean patients [10]. The classic dual-plane technique elevated the lateral border of the pectoralis major with partial inferior release, leaving the muscle tensioned over the upper third to half of the implant and an ADM sling supporting the inferolateral pole. The modified technique released the inferior margin completely, allowing the muscle to retract superiorly and drape over the upper pole without inferiorly directed pull, with a rectangular matrix sutured to the released margin. In every case, the pocket was irrigated with antibiotic solution, the implant was introduced through a funnel using a no-touch technique, final sizing was confirmed in the sitting position, and closed-suction drains were placed. Two-stage reconstruction placed a tissue expander at the mastectomy and exchanged it for a permanent implant after completion of expansion and of any adjuvant therapy. The operative record specified the plane in 177 of 633 implant-based procedures, leaving plane-level comparison beyond the reach of this series.

#### 2.3.2. Techniques of Autologous Reconstruction

The pedicled latissimus dorsi flap was raised on the thoracodorsal pedicle and tunneled through the axillary pathway [11]. Five designs were used according to the volume and the skin required. The extended design harvested the muscle together with the surrounding fatty envelope to reconstruct a whole breast after total mastectomy. The mini design harvested a limited muscle segment to fill a partial defect after breast-conserving surgery, entering the breast through a separate axillary incision. The vertical and boomerang designs varied the orientation of the skin paddle to match the mastectomy scar and to improve donor-site closure. The boomerang design was developed at this institution to obtain greater volume and projection than the classic extended flap allows, and has been described previously in both the immediate and the delayed setting [4]. Technical refinements of the axillary approach to the extended design have likewise been reported [12]. A minimally invasive variant was used during part of the study period. The muscle alone was elevated through a single mid-axillary incision, with endoscopic assistance from 2019 and with robotic assistance from 2020, and was transferred without a skin paddle, leaving no scar on the back. Volume was governed by the extent of muscle harvested, so that a partial harvest corresponds to the mini design and a maximal harvest to the extended design. Both approaches have been reported separately from this institution [5,6]. Reconstruction of this type was confined to partial mastectomy defects, since the volume obtainable from muscle alone is limited in Korean women. A small silicone implant was placed beneath the flap in patients for whom flap volume alone proved insufficient. Abdominal reconstruction used a pedicled, free, or delayed transverse rectus abdominis myocutaneous (TRAM) flap, and perforator reconstruction used a thoracodorsal artery perforator (TDAP) or deep inferior epigastric perforator (DIEP) flap; both remained uncommon, reflecting an institutional preference for pedicled thoracic donor sites.

#### 2.3.3. Techniques of Oncoplastic Breast-Conserving Surgery

Oncoplastic procedures were grouped by the established distinction between volume displacement and volume replacement [13,14], an approach adapted specifically to the small to moderate breast volumes prevailing in Korean women [15,16]. Volume-displacement techniques redistributed the residual parenchyma within the breast. Glandular reshaping mobilized and advanced glandular flaps to obliterate the lumpectomy cavity. The rotation flap rotated a de-epithelialized parenchymal flap into the defect. The tennis-racket method combined a circumareolar incision with a radial extension over the tumor, permitting resection and closure through a single incision. Therapeutic reduction mammoplasty or mastopexy resected the tumor within a reduction pattern, with contralateral symmetrization where indicated; comparable periareolar approaches have been reported in Korean practice [17]. Volume-replacement techniques imported tissue from outside the breast: the lateral thoracodorsal flap transferred a fasciocutaneous flap from the lateral chest wall, and the lateral intercostal artery perforator (LICAP) flap transferred a perforator-based flap from the same region with the muscle preserved, following the pedicled perforator principle described for breast reconstruction [18] and the intercostal perforator anatomy subsequently defined [19].

#### 2.3.4. Techniques of Chest Wall Reconstruction

Coverage of a chest wall defect used local and regional flaps, including thoracoabdominal designs, which remain the mainstay of closure in this setting [20,21]. The method was chosen for the size and the site of the defect and for the speed with which durable cover could be obtained: a latissimus dorsi flap of any design, a rotation or bilateral advancement flap, a local flap with a split-thickness skin graft, or direct closure were each used. No free tissue transfer was undertaken in this group, since systemic disease burden, previous irradiation of the recipient bed, and the need to resume oncological treatment without delay all weigh against prolonged microsurgery.

#### 2.3.5. Techniques for Adjunctive and Secondary Procedures

Nipple–areola reconstruction used a local C-V flap, a free nipple graft, or areolar tattooing, generally as a planned later stage. Contralateral balancing employed reduction mammoplasty, mastopexy, or augmentation. Fat grafting corrected contour irregularity. Revision surgery on an existing prosthesis comprised implant removal, implant exchange, capsulotomy, and capsulectomy.

### 2.4. Radiotherapy

Whole-breast irradiation was routinely delivered after breast-conserving surgery, whereas post-mastectomy radiotherapy (PMRT) was delivered according to disease stage and risk factors. Radiotherapy was classified throughout by its timing relative to the reconstruction rather than by its timing relative to the oncological resection, and the terms neoadjuvant and adjuvant are therefore not used for irradiation in this report. Prior irradiation denoted irradiation of the ipsilateral breast or chest wall during a previous, separate treatment course, so that the reconstruction was performed in a previously irradiated field. Radiotherapy delivered as part of the index treatment course was subdivided into pre-reconstruction radiotherapy and post-reconstruction radiotherapy. Pre-reconstruction radiotherapy denoted irradiation given before the reconstruction, either preoperatively, as for unresectable or inflammatory disease, or postoperatively when reconstruction was deferred until irradiation had been completed. Post-reconstruction radiotherapy denoted irradiation given after an immediate reconstruction. The remaining patients received no irradiation of the breast or chest wall. The database recorded irradiation only as preceding or following the reconstruction, so prior irradiation could not be separated from the preoperative component of pre-reconstruction radiotherapy; the two are counted together as pre-reconstruction radiotherapy throughout. A patient irradiated both before and after reconstruction is counted only in the pre-reconstruction category, so the categories are mutually exclusive.

The target volume was determined by stage and risk factors and, where necessary, by multidisciplinary discussion. Regional nodal irradiation was not routinely delivered as a comprehensive field; the axillary, supraclavicular, and internal mammary regions were included selectively according to risk. Conventional fractionation of 50 to 50.4 Gy in 25 to 28 fractions was the preferred regimen, and hypofractionated radiotherapy of 40 to 42.6 Gy in 15 to 16 fractions was introduced in the later years of the study. A boost, where indicated, delivered a further 10 to 16 Gy in 4 to 8 fractions.

### 2.5. Chemotherapy

Chemotherapy was likewise recorded as a timing category, namely neoadjuvant, adjuvant, or none, without agent, regimen, cycle number, or dose. The categories are mutually exclusive; a patient treated both before and after operation counts as neoadjuvant. Preoperative chemotherapy in a patient with M1 disease was classified as palliative systemic therapy rather than as neoadjuvant treatment, since the operation in those patients was not undertaken with curative intent. Neoadjuvant treatment was given for locally advanced disease or to permit breast conservation in patients who would otherwise have required mastectomy, and adjuvant treatment followed stage, receptor status, and multidisciplinary assessment. Three patients declined recommended chemotherapy and appear as untreated. Endocrine therapy and targeted agents were absent from the database and could not be analyzed, a limitation of consequence given their contribution to survival in hormone-receptor-positive and HER2-positive disease.

### 2.6. Outcome Assessment

Vital status was recorded in the institutional database by explicit annotation of patients known to be deceased. Mortality was confirmed against the institutional medical record, in which the terminal admission or the report of the terminal event is documented by the treating team; no linkage to an external death registry was available. Patients of uncertain vital status are exactly the 41 whose insurance eligibility had terminated, and the consequence of misclassifying that group as deceased is quantified in Section 3.9 and Section 4.5. A second, separately annotated group comprised patients whose National Health Insurance eligibility had terminated. In Korea, eligibility may lapse for several reasons, of which mortality is only one; emigration or prolonged residence abroad, transfer to the Medical Aid program, and loss of nationality all produce the same administrative signal. These patients were therefore not classified as deceased in the primary analysis. We instead treated the assumption as a testable hypothesis: if lapsed eligibility largely reflected mortality, the disease-burden profile of that group should resemble the confirmed deceased. The record of every patient so flagged was additionally interrogated for the last documented attendance at this institution, and the date of that attendance, its setting (outpatient clinic, inpatient admission, or emergency department), and the clinical department concerned were recorded. Attendance in person establishes that the patient was alive on the date shown and therefore places a lower bound on survival; the date on which eligibility lapsed was absent from the record, so the two events could not be ordered.

A date of the terminal event was retrieved for every deceased patient, permitting description of the interval from reconstruction to that event, and the cause of the terminal event was abstracted from the terminal admission record and classified as progression of breast cancer or as another cause. Dates of last contact for surviving patients remained unavailable, so no denominator of person-time could be constructed, and formal survival analysis stayed out of reach; mortality therefore appears as a binary status recorded at any time during follow-up, supplemented by the interval to the terminal event among the deceased.

### 2.7. Statistical Analysis

Continuous variables are summarized as mean ± standard deviation and median (interquartile range), categorical variables as counts and percentages, and proportions with Wilson 95% confidence intervals (CIs); the test used for each comparison is named with the corresponding result. Stage was normalized across Roman, Arabic, and full-width notations and collapsed into 0/I, II, and III/IV. A multivariable logistic regression model for recorded mortality included age, stage group, neoadjuvant chemotherapy, and reconstructive strategy as covariates; the number of covariates was deliberately restricted given the small number of events. The change in reconstructive practice was examined by segmented logistic regression at patient level, with the index reconstruction as the unit and time in years from the first operation of the series as the underlying scale. Three change points were specified in advance from documented external events rather than chosen from the data: reimbursement of post-mastectomy reconstruction by the National Health Insurance Service in April 2015, the withdrawal of textured implants in August 2019, and the recall of the sole domestically manufactured implant in November 2020. The model contained a baseline slope and one slope-change term for each change point, and implant-based and autologous reconstruction were modeled separately; the full specification and a sensitivity analysis adding a level shift at each change point are given in Table S2. Outcome analyses were conducted at the patient level, since repeated operations within a patient violate the independence assumption; the exception is the comparison of reoperation by radiotherapy status (Section 3.6), for which the reconstruction is the unit, since radiotherapy was recorded in relation to a given reconstruction. Procedure-level figures are otherwise reported descriptively only. Because follow-up ranged from a few weeks to thirteen years while the technique mix shifted over the same period, crude reoperation proportions conflate risk with opportunity for observation. Reoperation for a complication was therefore additionally analyzed at fixed time points of 90 days, one year, and three years after the index operation; for each window, the denominator was restricted to patients whose index operation preceded the end of the observation period by at least the length of that window, so that every patient counted had the full window of potential observation. A propensity-score analysis and a more extensively adjusted mortality model were considered and not undertaken, since the 29 events available for complete-case regression already constrain the four-covariate model, and neither comorbidity nor recurrence was captured in the database. Two-sided *p* < 0.05 was considered significant. Analyses were performed in IBM SPSS Statistics version 31.0 (IBM Corp., Armonk, NY, USA).

## 3. Results

### 3.1. Baseline Characteristics of the Cohort

Two thousand two hundred sixteen patients underwent 2523 reconstructive procedures over the thirteen-year period, yielding 2900 individually coded technique components. Mean age at the index operation was 47.6 ± 8.5 years (median 47, IQR 42–52; range 14–85) and mean body mass index (BMI) was 23.1 ± 3.4 kg/m^2^, with 536 patients (24.2%) at or above 25 kg/m^2^. Total mastectomy and partial mastectomy were almost equally represented (1042 [47.0%] and 1029 [46.4%], respectively). Invasive ductal carcinoma accounted for 1293 patients (58.3%) and ductal carcinoma in situ for 514 (23.2%). Neoadjuvant chemotherapy had been given to 263 patients (11.9%), a further 12 (0.5%) received preoperative chemotherapy classified as palliative systemic therapy for M1 disease, and post-reconstruction radiotherapy had been given to 967 (43.6%). Table 1 presents the baseline characteristics.

### 3.2. The Reconstructive Spectrum

Table 2 sets out the distribution of techniques by reconstructive intent at both procedure and patient level, together with the patient and disease profile associated with each technique. Restorative procedures accounted for 2094 patients (94.5%) and coverage procedures for 14 (0.6%), with the remainder adjunctive.

#### 3.2.1. Implant-Based Reconstruction

Direct-to-implant reconstruction was the single most common operation in the series (529 procedures; 504 patients as the primary technique). It was performed almost exclusively after total mastectomy (98.3%) in the most oncologically favorable population of the mastectomy groups: 33.9% had stage II or higher disease, 8.5% had received neoadjuvant chemotherapy, and only 15.9% went on to post-reconstruction radiotherapy. Two-stage expander reconstruction was performed in a smaller and more advanced group (58 patients; stage II or higher in 63.9%, neoadjuvant chemotherapy in 20.7%). Expander-to-implant exchange contributed a further 45 procedures.

#### 3.2.2. Latissimus Dorsi Flap-Based Reconstruction

The latissimus dorsi flap was used in five distinct designs that together accounted for 855 patients (856 including one patient whose design could not be assigned to any of the five), and the indications for these designs diverged sharply. The extended design (419 procedures; 405 patients) served whole-breast reconstruction after total mastectomy in 87.0% of cases and carried the heaviest oncological burden of the autologous group, with stage II or higher disease in 50.7% and neoadjuvant chemotherapy in 21.0%. The mini design (287 procedures; 284 patients) served the opposite purpose: total mastectomy accounted for only 1.4% of these cases, and 68.3% received post-reconstruction radiotherapy. The vertical design (58 procedures; 57 patients) followed the same conservation-oriented pattern, with no case following total mastectomy and 68.4% receiving post-reconstruction radiotherapy. The boomerang design (55 procedures; 52 patients), an institutional modification of the extended flap [4], reverted to the mastectomy setting (90.0%), and combination of a latissimus dorsi flap with an implant (64 procedures; 57 patients) was likewise a mastectomy technique (98.2%) used where flap volume alone was insufficient.

#### 3.2.3. Abdominal Flap-Based Reconstruction

Abdominal-based reconstruction was uncommon and declined to near-disappearance: 50 abdominal-flap procedures were performed in 48 patients—33 pedicled TRAM, 12 muscle-sparing free TRAM, and five DIEP—falling from 39 patients in 2012–2017 to 10 in 2018–2021 and a single patient in 2022–2026. Recipients of an abdominal flap had the highest BMI among the flap groups (25.3 ± 3.5 kg/m^2^), rising across the three variants from 24.6 ± 3.4 kg/m^2^ for pedicled TRAM to 25.9 ± 3.7 for muscle-sparing free TRAM and 27.7 ± 3.4 for DIEP. Every abdominal flap in which the mastectomy extent was recorded followed a total mastectomy, and 28.0% had received neoadjuvant chemotherapy. Free tissue transfer remained infrequent throughout: the abdominal donor site was used microsurgically in 16 patients (12 muscle-sparing free TRAM and four DIEP), and thoracic perforator flaps were confined to three TDAP flaps.

#### 3.2.4. Oncoplastic Breast-Conserving Surgery

Six recognizable oncoplastic techniques were used in 623 patients. Glandular reshaping was the commonest (235 procedures; 188 patients), followed by rotation flap (156; 151), therapeutic reduction or mastopexy (151; 117), lateral thoracodorsal flap (114; 111), tennis-racket resection (39; 38), and LICAP flap (18; 18). Total mastectomy accounted for under 5% of each of these groups, and post-reconstruction radiotherapy was correspondingly frequent, reaching 86.8% after tennis-racket resection and 71.2% after lateral thoracodorsal flap. Body mass index was highest in the therapeutic reduction or mastopexy group (26.2 ± 4.5 kg/m^2^). Volume-displacement techniques such as glandular reshaping and rotation flap grew over the study period, from 23 and 60 patients, respectively, in 2012–2017 to 116 and 59 in 2022–2026.

#### 3.2.5. Adjunctive and Secondary Procedures

Adjunctive procedures formed a substantial share of operative workload while rarely constituting a patient’s primary technique. Revision surgery on an existing prosthesis, comprising implant removal, exchange, and capsule revision surgery, accounted for 186 procedures yet was the primary technique in only 43 patients; this group was the oldest in the series apart from the chest wall group (55.9 ± 9.5 years) and had essentially no neoadjuvant treatment. Contralateral balancing accounted for 146 procedures, nipple–areola reconstruction for 83, and fat grafting or liposuction for 25.

### 3.3. Changing Practice over Thirteen Years

Practice changed substantially over the study period (Figure 1). In 2014, autologous reconstruction accounted for 67 of 114 index procedures (58.8%) and implant-based reconstruction for four (3.5%). By 2026, the position had reversed: autologous reconstruction accounted for 15 of 160 index procedures (9.4%) and implant-based reconstruction for 70 (43.8%). Within the autologous group, the extended latissimus dorsi flap fell from 173 patients in 2012–2017 to 54 in 2022–2026, whereas the boomerang and vertical designs, absent before 2018, together accounted for 74 patients in the final era. Oncoplastic breast-conserving surgery persisted throughout without a consistent trend, its share of the annual caseload fluctuating between roughly an eighth and a half.

Delayed reconstruction was uncommon yet showed the clearest donor-site shift in the series. Thirty-three procedures were explicitly recorded as delayed. Every one of the 13 performed in 2012–2017 used an abdominal flap, as did seven of the nine performed in 2018–2021; none of the 11 performed in 2022–2026 used one (*p* < 0.001, Fisher exact test). The last delayed abdominal flap was carried out in November 2019. From 2021, the same indication was met with a latissimus dorsi flap, including the boomerang design in two cases, and from 2023 increasingly with a delayed expander-based reconstruction, which accounted for seven of the 11 procedures in the final era (Figure 2). These proportions rest on small denominators and should be read as counts rather than as rates. No delayed reconstruction in the thirteen years was performed as a direct-to-implant procedure.

Segmented logistic regression tested whether the three external events named in advance coincided with a change in the rate of adoption (Figure S1, Table S2). Before April 2015, the implant-based share rose without reaching significance (odds ratio 2.69 per year, 95% CI 0.84–8.60), and reimbursement did not detectably alter that slope (change in slope *p* = 0.30), the implant share continuing to rise at 1.41 per year (1.24–1.60) through to 2019. The withdrawal of textured implants marked the one unambiguous interruption in the series: the slope reversed, the odds of an index reconstruction being implant-based falling by 0.28 per year (0.20–0.39), a change in slope corresponding to an odds ratio of 0.20 per year (0.13–0.30, *p* < 0.001), and the implant share fell from 34% to 10% over the following fourteen months. The domestic implant recall did not extend that fall. The slope reversed again from November 2020 (1.58 per year, 1.46–1.70; change in slope 5.73, 3.86–8.51, *p* < 0.001), and the implant share recovered to exceed its pre-2019 level by 2025. Autologous reconstruction mirrored this pattern, with its decline arrested at reimbursement (change in slope 2.69, 1.52–4.77, *p* = 0.001) and resuming from November 2020 (0.40, 0.29–0.56, *p* < 0.001). Adding a level shift at each change point improved fit (likelihood-ratio *p* = 0.001) without altering the direction or the ordering of the slope changes. These estimates describe an uncontrolled single-center series and identify coincidence in time rather than cause.

Minimally invasive harvest of the latissimus dorsi muscle formed a distinct episode within the same period. Forty-one procedures elevated the muscle alone through a single mid-axillary incision, 24 with endoscopic and 17 with robotic assistance. Use rose from 12 procedures in 2019 to a peak of 15 in 2020, fell to 10 in 2021, and amounted to one procedure in 2022 and three in 2023. Thirty-three were partial harvests corresponding to the mini design, and eight were maximal harvests corresponding to the extended design.

### 3.4. Reconstructive Strategy and Disease Burden

The four strategies served demonstrably different populations (Table 3). Patients undergoing chest wall reconstruction were on average fifteen years older than those undergoing autologous reconstruction (61.6 ± 11.3 vs. 46.4 ± 7.3 years; Kruskal–Wallis *p* < 0.001), and two-thirds of those with a recorded stage had stage III or IV disease, compared with 10.1% of the autologous group, 8.8% of the implant group, and 4.5% of the oncoplastic group (*p* < 0.001). Neoadjuvant chemotherapy had been given to 42.9% of the chest wall group, compared with 8.7–15.9% elsewhere. The implant-based group represented the most oncologically favorable population undergoing mastectomy, and the oncoplastic group most often went on to post-reconstruction radiotherapy (64.4%).

### 3.5. Reoperation Burden

Of the 2216 patients, 1976 (89.2%) underwent a single operation and 240 (10.8%) underwent a further 307 operations, giving 2523 operations in total. The median interval to a further operation was 378 days, and 12% of these operations occurred within 90 days of the index procedure. The 307 repeat operations were classified by their principal indication and comprised 98 whose principal indication was a complication (31.9%), 47 staged completions of a two-stage prosthetic reconstruction (15.3%), 42 nipple–areola reconstructions (13.7%), 29 new reconstructions for metachronous contralateral disease or recurrence (9.4%), 12 contour refinements (3.9%), five contralateral balancing procedures (1.6%), and 74 of mixed indication (24.1%). Counted at patient level, and including operations of mixed indication in which a complication formed one component, 101 patients (4.6%) underwent at least one reoperation for a complication (Table 4).

Reoperation burden differed markedly by primary technique (Table 4). Two-stage expander reconstruction required 2.22 operations per patient by design, and 13.8% of these patients underwent an additional operation for a complication, a figure statistically indistinguishable from the 10.5% recorded after direct-to-implant reconstruction. Direct-to-implant reconstruction required 1.22 operations per patient, and latissimus dorsi flap combined with an implant reached the highest rate in the series (10 of 57 patients, 17.5%, 95% CI 9.8–29.4), a proportion whose confidence interval overlaps those of direct-to-implant (10.5%, 8.1–13.5) and two-stage expander reconstruction (13.8%, 7.2–24.9). Purely autologous and oncoplastic techniques were substantially more stable: the mini latissimus dorsi flap required 1.05 operations per patient with a complication reoperation rate of 1.4%, and the lateral thoracodorsal flap required 1.01 operations per patient with no complication reoperation recorded.

At fixed time points, chosen to remove the dependence of crude proportions on unequal observation time, reoperation for a complication had occurred by 90 days in 8/533 (1.5%) patients after implant-based reconstruction, 9/900 (1.0%) after autologous reconstruction, and 1/588 (0.2%) after oncoplastic surgery; by one year in 34/443 (7.7%), 15/882 (1.7%), and 0/540 (0.0%); and by three years in 22/295 (7.5%), 19/831 (2.3%), and 1/452 (0.2%). Each denominator is restricted to patients whose index operation preceded the end of observation by at least the corresponding interval, so that every patient counted had the full window of potential observation; deceased patients remained in the denominator. Among irradiated prosthetic reconstructions, the corresponding one-year figures were 8/85 (9.4%) after direct-to-implant reconstruction and 1/20 (5.0%) after two-stage expander insertion.

### 3.6. Oncological Treatment in Relation to Reconstruction

Radiotherapy and chemotherapy were recorded for 1975 (89.1%) and 1986 patients (89.6%), respectively, and both were documented as binary variables without dose, fractionation, or agent. Among the 1953 patients with both recorded, 539 (27.6%) received neither modality, 471 (24.1%) received both adjuvant chemotherapy and post-reconstruction radiotherapy, and 164 (8.4%) received neoadjuvant chemotherapy followed by post-reconstruction radiotherapy. Use of both modalities rose with stage: neoadjuvant chemotherapy was given to 6.7% of patients with stage 0/I disease, 14.6% with stage II, and 51.0% with stage III/IV; adjuvant chemotherapy to 30.9%, 65.6%, and 40.6%; and post-reconstruction radiotherapy to 47.3%, 54.5%, and 58.7%, respectively.

Use differed profoundly by reconstructive strategy (Table 2 and Table 3). Post-reconstruction radiotherapy followed 64.4% of oncoplastic breast-conserving procedures and 45.6% of autologous reconstructions, yet only 17.9% of implant-based reconstructions (*p* < 0.001). The gradient was similarly steep across individual techniques, ranging from 86.8% after tennis-racket resection and 71.2% after a lateral thoracodorsal flap down to 15.9% after direct-to-implant reconstruction. Adjuvant chemotherapy was distributed far more evenly, reaching 42.8% of autologous and 42.3% of oncoplastic reconstructions against 30.1% of implant-based reconstructions, and ranging across techniques from 27.1% after two-stage expander insertion to 59.5% after tennis-racket resection.

Reoperation for a complication after irradiation is set out in Table 5. Among implant-based reconstructions, patients who received post-reconstruction radiotherapy underwent reoperation for a complication about twice as often as the remainder (21 of 110, 19.1%, vs. 42 of 451, 9.3%; *p* = 0.006), and the same contrast held within direct-to-implant reconstruction considered alone (18.0% vs. 9.2%; *p* = 0.023). Autologous reconstruction showed no such difference (2.6% vs. 3.5%; *p* = 0.45).

The two prosthetic strategies were also compared directly. Among irradiated patients, reoperation for a complication followed 16 of 89 direct-to-implant reconstructions (18.0%) and five of 21 expander-based reconstructions (23.8%), a difference far from significance (Fisher exact test, *p* = 0.55); the same held among patients not irradiated after reconstruction (9.2% vs. 7.9%; *p* = 1.00) and across the whole prosthetic cohort (10.8% vs. 13.6%; *p* = 0.51). Irradiated and non-irradiated implant recipients nevertheless differed substantially in disease burden (Table 5). Neither the reconstructive plane nor the delivery of a sequential chest wall boost was recorded consistently.

### 3.7. Chest Wall Reconstruction

Fourteen patients underwent chest wall reconstruction for coverage rather than for restoration of breast form (Table 6). Every patient in this group with a recorded stage had stage II or higher disease, and none had stage 0/I disease, against 50.1% stage 0/I in the autologous reconstruction group. Neoadjuvant chemotherapy had been given to 42.9%, and mean age was 61.6 ± 11.3 years. The resection was full thickness in every instance: total or modified radical mastectomy with wide excision of the overlying skin envelope preceded eleven of these procedures, one was documented as a wide excision carried out together with chest wall resection, and the oncological procedure was not recorded in the remaining two. The extent of resection was coded in 11 patients in the source dataset, 10 of whom (90.9%) were recorded as total mastectomy; the remaining case was documented by the oncological team as a mastectomy, so all 11 were in fact full-thickness resections.

Coverage was achieved with a bilateral advancement flap in four patients and an extended latissimus dorsi flap in three, and with a vertical latissimus dorsi flap, a boomerang latissimus dorsi flap, a mini boomerang latissimus dorsi flap, a thoracoabdominal flap, a local flap with split-thickness skin graft, a rotation flap, and primary closure in one each. Four patients (28.6%) were deceased.

### 3.8. Mortality

Mortality was recorded in 39 of 2216 patients (1.8%). On univariable analysis, mortality rose with stage (0.6% for stage 0/I, 2.3% for stage II, 6.3% for stage III/IV; *p* < 0.001), and was higher in patients who had received neoadjuvant chemotherapy (6.8% vs. 1.1%; *p* < 0.001) and pre-reconstruction radiotherapy (15.6% vs. 1.6%; *p* < 0.001). Two of the twelve patients whose preoperative chemotherapy was classified as palliative systemic therapy for M1 disease were deceased (16.7%; *p* = 0.008). BMI was marginally higher in the deceased (24.4 ± 4.0 vs. 23.1 ± 3.4 kg/m^2^; *p* = 0.051). Age (*p* = 0.25), adjuvant chemotherapy (*p* = 0.92), post-reconstruction radiotherapy (*p* = 0.62), and operating surgeon (*p* = 0.47) showed no association with mortality (Table 7, univariable comparison).

The cause of the terminal event was retrieved for every deceased patient (Table 8). Thirty-seven of the 39 followed progression of breast cancer, two of them in patients with a documented concurrent second primary. The remaining two were not attributable to breast carcinoma: one patient had a metastatic malignant phyllodes tumor and one a metastatic pancreatic carcinoma more than six years after reconstruction. Breast-cancer-specific mortality was therefore 37 of 2216 patients (1.7%).

Recorded mortality differed across individual techniques (Figure 3). No death was recorded after glandular reshaping (0/188), tennis-racket resection (0/38), the vertical latissimus dorsi design (0/57), or two-stage expander insertion (0/58); mortality was 0.4% after direct-to-implant reconstruction (2/504), 5.3% after a latissimus dorsi flap combined with an implant (3/57), and 28.6% after chest wall reconstruction (4/14).

In the multivariable model (Table 7, multivariable model 1; 1636 patients, 29 events), stage II disease (odds ratio 3.56, 95% CI 1.28–9.96) and stage III/IV disease (5.25, 1.53–18.07) showed independent associations with mortality, and neoadjuvant chemotherapy fell at the margin of significance (2.34, 0.99–5.50). Relative to autologous reconstruction, chest wall reconstruction carried higher odds of mortality (14.43, 1.84–113.00) and implant-based reconstruction carried lower odds (0.22, 0.05–0.97); the estimate for oncoplastic surgery pointed in the same direction without reaching significance (0.37, 0.12–1.12). The confidence intervals are wide, and the model explained a modest proportion of variance (pseudo-R^2^ 0.140). A sensitivity analysis omitting the coverage-intent group left the remaining estimates essentially unchanged (implant-based 0.23, 0.05–0.98; oncoplastic 0.40, 0.13–1.19; neoadjuvant chemotherapy 2.70, 1.14–6.43). The model was also refitted without neoadjuvant chemotherapy, which is partly collinear with stage (Table 7, multivariable model 2). The explanatory weight moved to stage, whose odds ratios rose to 3.96 (1.43–10.95) for stage II and 7.35 (2.26–23.87) for stage III/IV, while the estimates for reconstructive strategy were unchanged in direction and rank order (implant-based 0.22, 0.05–0.94; oncoplastic 0.34, 0.11–1.01; chest wall reconstruction 16.11, 2.12–122.63).

Restricting the outcome to the 37 patients whose terminal event was attributed to breast cancer left the gradient across strategies intact: 0.4% after implant-based reconstruction (2/564), 0.8% after oncoplastic surgery (5/623), 2.8% after autologous reconstruction (25/907), and 21.4% after chest wall reconstruction (3/14), against 0.4%, 0.8%, 2.9%, and 28.6% for all-cause mortality. The gradient with stage was likewise preserved (0.6%, 2.2%, and 6.3% for stage 0/I, II, and III/IV).

A date was available for all 39 deceased patients. The median interval from the index reconstruction to the recorded mortality event was 979 days (2.7 years; IQR 373–1516; range 75–3835). The event occurred within one year of the index operation in ten patients, between one and three years in twelve, between three and five years in nine, and beyond five years in eight (Figure 4). The interval tracked disease burden as closely as the occurrence of the event itself. The median interval fell from 1490 days in patients with stage 0/I disease to 1163 days in stage II and 345 days in stage III/IV. Among patients who had received neoadjuvant chemotherapy, the median interval was 552 days against 1286 days for those who had not (*p* = 0.002). The coverage-intent group stood apart most sharply: three of the four events after chest wall reconstruction occurred at 75, 77, and 132 days, so that the median for the group as a whole was 104 days against 1016 days for every other category combined. The fourth occurred at 2252 days and was attributable to pancreatic carcinoma.

### 3.9. Eligibility-Terminated Patients Compared with the Deceased and with the Remaining Cohort

Forty-one patients were flagged as having lost National Health Insurance eligibility. They differed markedly from the 39 confirmed deceased (Figure 5, Table 9). Neoadjuvant chemotherapy had been given to 14.6% of the eligibility-terminated group compared with 46.2% of the deceased (*p* = 0.0031), and 43.9% had stage 0/I disease compared with 12.8% of the deceased (*p* = 0.0028). No patient in the terminated group had received pre-reconstruction radiotherapy or had undergone chest wall reconstruction. Compared instead with the 2136 remaining patients, the terminated group proved statistically indistinguishable on every measure examined: neoadjuvant chemotherapy 14.6% vs. 11.2% (*p* = 0.45), stage III/IV 7.3% vs. 6.1% (*p* = 0.74), autologous reconstruction 48.8% vs. 40.3% (*p* = 0.34), and mean age 48.0 vs. 47.6 years. The eligibility-terminated group also clustered in the earlier years of the series, with 87.8% having had their index operation before 2021.

The last documented attendance at this institution was retrieved for all 41 patients (Table 10). Every patient had attended in person after the index reconstruction, at a median of 4.3 years afterwards (IQR 1.8–7.5; range 0.4–10.6), an interval longer than that from reconstruction to the terminal event among the deceased (median 2.7 years, IQR 1.0–4.2; *p* = 0.017, Mann–Whitney). The attendance was an outpatient consultation in 35 patients, an inpatient admission in four, and an emergency department visit in two. Fifteen patients (36.6%) last attended during 2024 or later and five during 2026, the final year of the series, so those patients were alive on dates at which an eligibility lapse arising from death could not yet have occurred. Twenty-six of the final attendances were at departments involved in breast cancer care, namely medical oncology in 13, breast and thyroid surgery in seven, plastic and reconstructive surgery in five, and nuclear medicine in one; the remaining 15 were at departments with no part in breast cancer follow-up, namely obstetrics and gynecology in four, endocrinology, gastroenterology, psychiatry, and emergency medicine in two each, and family medicine, neurosurgery, and neurology in one each. Three patients were documented as transferred to another institution at that final attendance.

## 4. Discussion

This series describes thirteen years of reconstructive breast surgery at a single Korean tertiary center. Three findings deserve emphasis.

First, the reconstructive spectrum is considerably wider than administrative data can represent. Twenty-six distinct techniques were required to describe 2523 operations, and the five latissimus dorsi designs alone accounted for more patients than any other single approach. National claims-based analyses have proved valuable in documenting the growth of reconstruction in Korea after insurance coverage began in 2015 [2,3]; they classify by reimbursement code, however, and therefore merge a mini latissimus dorsi flap used to fill a lumpectomy defect with an extended flap used to reconstruct a whole breast. Our data quantify how misleading that merger becomes: the mini design followed total mastectomy in 1.4% of cases and the extended design in 87.0%; the two operations belong to essentially disjoint clinical populations despite sharing a donor muscle. They also differ in donor-site morbidity, operative time, and esthetic objective. Institutional series that report their classification transparently therefore remain necessary complements to registry work.

Second, our data reproduce the expected national shift toward implant-based reconstruction while revealing something the national data omit: a large and stable oncoplastic workload. National reconstruction statistics are indexed to mastectomy; volume-displacement and volume-replacement procedures performed alongside breast conservation therefore remain largely invisible within them. In our series, oncoplastic breast-conserving surgery accounted for 623 patients (28.1%), a caseload comparable to implant-based reconstruction, consistent with evidence that oncoplastic techniques permit wider resection without compromising the conservation approach [22]. Any assessment of reconstructive service provision that counts only post-mastectomy procedures will therefore substantially understate the reconstructive burden.

Third, and most importantly for interpretation, reconstructive modality showed a strong association with mortality, and this association is almost certainly non-causal. The gradient runs precisely as disease burden runs: chest wall reconstruction, performed for locally advanced or recurrent disease, carried 28.6% mortality; autologous reconstruction, preferred in patients with larger resections and greater adjuvant treatment requirements, carried 2.9%; implant-based and oncoplastic surgery, offered to the most favorable patients, carried 0.4% and 0.8%. The associations persisted after adjustment for stage and neoadjuvant chemotherapy; this persistence, however, reads better as evidence of residual confounding than as evidence of effect, since stage was missing in 24.2% of patients and the recorded covariates fail to capture the surgical judgment that determined technique in the first place. Nor is disease burden the only input to that judgment: reconstructive choice also depends on anatomy and donor-site availability—a patient without adequate abdominal donor tissue cannot receive an abdominal flap whatever her oncological situation—so the strategy variable summarizes constitutional as well as oncological selection, and neither is captured fully by the recorded covariates. The two model specifications redistribute the same signal between neoadjuvant chemotherapy and stage without altering the ordering of the reconstructive categories, which indicates that the strategy variable carries disease burden rather than any effect of the operation. This represents confounding by indication in a clean form, and it carries a practical warning. Comparative studies of reconstructive technique that report oncological outcomes without full adjustment for the indications driving technique selection will systematically favor whichever technique is offered to healthier patients. In the current era, that technique is the implant.

The same reasoning applies with equal force to the oncological treatment variables, and the point deserves explicit statement because the direction of the association invites misreading. Neoadjuvant chemotherapy carried an adjusted odds ratio of 2.34 for recorded mortality, and irradiation preceding the reconstruction was associated with a mortality of 15.6% against 1.6%. Neither figure indicates that these treatments are harmful. Neoadjuvant chemotherapy is given at this institution for locally advanced disease, or to make breast conservation possible in a patient who would otherwise require mastectomy; it is therefore an indicator of tumor size, nodal involvement, and aggressive biology rather than a treatment that patients with otherwise equivalent disease either receive or avoid. Randomized comparisons of the same regimens delivered before and after surgery have shown no difference in distant recurrence, breast cancer mortality, or all-cause mortality [23], so the excess mortality observed here belongs to the disease that prompted the treatment rather than to the treatment itself. Neoadjuvant chemotherapy rose steeply with stage, from 6.7% in stage 0/I to 51.0% in stage III/IV, and removing it from the model transferred its explanatory weight onto stage rather than dissipating it. Operation in a previously irradiated field requires even greater caution. Only 32 patients (1.4%) were irradiated before reconstruction, and in Korean practice, irradiation delivered before rather than after the resection is reserved for a narrow set of indications, principally unresectable locally advanced disease, inflammatory carcinoma, and chest wall recurrence. The variable therefore functions as a marker of salvage or palliative intent rather than as a therapeutic intervention, and the crude figure of 15.6% rests on five events among 32 patients. Pre-reconstruction radiotherapy was for that reason not entered into the multivariable models. Post-reconstruction radiotherapy, delivered to 43.6% of the cohort in the conventional postoperative setting, showed no association with mortality at all (*p* = 0.62). Every association reported here between a treatment and mortality should accordingly be read as prognostic rather than causal.

### 4.1. Rationale for a Latissimus Dorsi-Based Autologous Strategy

Abdominal donor sites dominate autologous breast reconstruction in many Western centers, and the muscle-sparing free TRAM and the DIEP flap are widely treated as the reference standard. The present series runs in the opposite direction: abdominal flaps accounted for 50 of 2523 procedures (2.0%) and fell to a single patient in the most recent era, while latissimus dorsi-based reconstruction became the dominant autologous technique. This reflects a considered institutional position rather than a lack of microsurgical capacity, and it rests on four arguments.

First, breast volume in a Korean population is generally modest. The median measured breast volume in this cohort was 290 mL (IQR 220–400), and 51.1% of all measured breasts fell below 300 mL. The principal advantage of an abdominal flap, the ability to transfer a large and reliable soft-tissue volume, is therefore seldom the limiting consideration, a point already established for oncoplastic practice in this population [15,16]. Second, the latissimus dorsi flap is pedicled. Avoiding a microvascular anastomosis shortens operative time and removes the specific hazard of anastomotic failure altogether; partial flap compromise after a pedicled transfer does not carry the requirement for emergent take-back that governs free tissue transfer. Third, a small implant can be placed beneath the flap where flap volume alone proves insufficient. This combination was used in 64 procedures and extends the volume range of the pedicled option well into the territory that would otherwise require an abdominal flap, while retaining the vascularized autologous cover that makes the construct tolerant of radiotherapy. Fourth, and historically the strongest indication for abdominal transfer at this institution, delayed reconstruction requires a large cutaneous paddle to rebuild the breast envelope rather than volume alone. TRAM and DIEP flaps were used in delayed cases even for small breasts because the abdomen supplied an area of skin that the conventional latissimus dorsi designs could not. The boomerang modification removed that constraint by reorienting the skin paddle to deliver a substantially larger transferable skin area, and its adoption maps directly onto the decline of abdominal transfer: the boomerang design was absent before 2018, was used in eight procedures during 2018–2021 and in 47 during 2022–2026, over precisely the period in which abdominal flaps fell to a single case.

A conceptual change has reinforced the same trend. Reconstruction here is no longer aimed at reproducing the preoperative volume and shape of the affected breast; the target is an ideal volume and shape agreed with the patient [24]. A large native breast once mandated a large flap, and the abdomen was often the only donor site able to supply it; a large breast is now more often managed by combining a moderate-volume reconstruction with a contralateral reduction or mastopexy, performed in 146 procedures in this series. These arguments are institutional and largely qualitative. Operative time, length of stay, donor-site morbidity, implant requirement, and patient satisfaction were not captured systematically in this database, so the comparison with abdominally based reconstruction cannot be made quantitative here; the prospective comparison of donor-site scar satisfaction among harvest approaches reported from this service [6] is the only patient-reported measure available.

These findings are not presented as evidence that abdominal free flaps should be abandoned. They remain indispensable where the thoracodorsal system is unavailable, where the latissimus has been divided or irradiated, and where a very large volume must be replaced without a contralateral procedure. What these data support is the narrower claim that in a population with predominantly small-to-moderate breast volume, a pedicled latissimus dorsi-based strategy can absorb almost the entire reconstructive caseload, including the delayed cases that previously drove abdominal transfer. Every delayed reconstruction up to 2019 used an abdominal flap, and none afterward did (Figure 2); the indication was met instead by the boomerang and other extended latissimus dorsi designs, which supply the skin paddle directly, and by delayed expander-based reconstruction, which creates the envelope by expansion.

### 4.2. External Influences on Technique Selection

Technique selection over this period was also shaped by external changes, marked on Figure 1. Reimbursement came first: from April 2015 the Korean National Health Insurance covered breast reconstruction after total mastectomy, and covered the ADM used to support it, so that the choice of technique ceased to be governed by what the patient could afford. Prepectoral placement followed, the first such direct-to-implant reconstruction being performed here in February 2017; removing the need to elevate pectoralis major made single-stage implant reconstruction tolerable for patients who would previously have been offered a flap or a two-stage expander. Large-format ADM, adopted from March 2021 in 16 × 16, 18 × 18, and 20 × 20 cm sheets, then made complete prosthetic coverage practicable in a single piece. The point is not that any one of these determined practice, rather that the menu from which surgeon and patient choose has broadened considerably.

Donor-site scarring drove a further line of development. Endoscopic elevation of the muscle alone through a single mid-axillary incision was introduced here in 2019 to remove the back scar altogether [5], and robotic elevation followed in 2020 [6]. A prospective comparison from this service found donor-site scar satisfaction higher after both approaches than after the conventional harvest, with no difference between them [6]. Neither displaced conventional harvest; its use peaked in 2020 and had largely ceased by 2022, constrained by an operating time some two hours longer, by competition for robotic capacity, and by a muscle-only flap supplying volume sufficient for a partial defect rather than for whole-breast reconstruction in a Korean population.

Two supply shocks reshaped prosthetic practice directly. In July 2019, the textured Allergan Natrelle devices were recalled worldwide because of breast implant-associated anaplastic large cell lymphoma (BIA-ALCL); the first Korean case was identified on 14 August 2019 [25], and another on 29 August 2019. The Ministry of Food and Drug Safety asked all domestic institutions to stop using textured implants of every manufacturer. The effect on our practice was immediate and complete. Textured anatomical devices, which accounted for 46 of the 56 implants recorded in 2017 and 21 of 72 in 2019, fell to two in 2020 and to none from 2021 onward, while smooth round devices rose from essentially zero before 2019 to 24 in 2021 and between 55 and 82 annually thereafter. This was not a neutral substitution. Textured shells could be manufactured as anatomical teardrop devices and as ellipses whose horizontal and vertical diameters differed, so that the implant itself could be selected to match the height and width of the breast footprint. Once only smooth round devices were available, every implant had the same diameter in all directions, and shape had to be created by the surrounding envelope rather than chosen off the shelf; the characteristic penalty is a step-off or depression at the upper pole. The rise in subpectoral placement in our later years should be read against this. It does not represent a return to the classic submuscular pocket, rather the adoption of a modified technique in which pectoralis major is used selectively to cover the upper pole of a round implant and to camouflage that depression deformity, while the remainder of the device sits in the prepectoral plane. The considerations governing this choice between planes have been set out separately by our group [7]. Prepectoral placement remains the default; the modified subpectoral pocket is a shape-driven exception created by the loss of anatomical implants.

A second shock followed within fifteen months. In November 2020, the Ministry of Food and Drug Safety ordered the suspension of sale and the recall of BellaGel, the only silicone breast implant manufactured domestically, after establishing that unapproved raw materials had been used in production since December 2015. The enforcement action was taken for a licensing violation rather than for any demonstrated harm. Testing of the finished devices by the regulator detected none of the toxic degradation products that had been feared; four of the five undeclared materials were components already licensed for other implantable devices in Korea; and an expert panel concluded that the product posed no safety problem for women who had already received it, with subsequent comparative series showing no worse performance than competitors [26]. Explantation was not recommended on safety grounds. Practice moved nonetheless, not because the device was shown to be unsafe, but rather because confidence in the prosthetic option was disturbed while the question remained open. In our own record, BellaGel accounted for 48 of the 72 implants used in 2019 and 53 of 69 in 2020, and disappeared entirely from 2021. The segmented regression qualifies this account in one important respect. Of the three events named in advance, only the withdrawal of textured implants behaved as an interruption in the statistical sense, reversing the adoption slope and depressing the implant share from 34% to 10% within fourteen months. The recall coincided with recovery rather than with further decline, so the attitudinal effect described here operated on the choice of device and on the willingness to revise an existing prosthesis rather than on the decision to reconstruct with a prosthesis at all. Two consequences followed. Some women already carrying a recalled device elected removal or exchange, adding a stream of unplanned revision surgery that was neither oncological nor reconstructive in intent. The more durable consequence was attitudinal: a second national recall within little more than a year left both surgeons and patients markedly more cautious about prosthetic reconstruction in general, and made an autologous or flap-assisted option easier to propose and easier to accept.

Patient-side factors have moved in parallel. National Health Insurance coverage in Korea is supplemented by a high prevalence of private indemnity insurance, so out-of-pocket cost has become a far weaker determinant of what is chosen than it was at the start of this period. Earlier detection and the correspondingly favorable survival of breast cancer have changed what is asked of reconstruction as well: with decades of survivorship in prospect, the durability of the result, the speed of return to ordinary activity, and the symmetry, size, and shape of the reconstructed breast all carry more weight than they did when the operation was regarded principally as restoration of contour. Two observations in our data are consistent with this account. Contralateral balancing procedures rose more than tenfold across the study period, from 0.7% of index reconstructions in 2012–2017 to 3.2% in 2018–2021 and 11.0% in 2022–2026 (*p* < 0.001), indicating that symmetry is increasingly pursued actively rather than accepted as whatever the reconstructed side happens to deliver. The age range considered suitable for reconstruction also widened at its upper end. Mean age at index reconstruction rose from 45.5 ± 7.8 years in 2012–2017 to 47.3 ± 8.1 in 2018–2021 and 49.4 ± 9.2 in 2022–2026 (*p* < 0.001), driven by the upper tail rather than by any loss of younger patients: the proportion aged 60 years or over rose from 2.6% to 14.2% (*p* < 0.001), those aged 65 or over from 0.9% to 5.9% (*p* < 0.001), and the oldest patient undergoing reconstruction from 74 to 85 years. Improvements in general medical care mean that women in their sixties, and selected women in their early seventies, are now fit enough to tolerate immediate reconstruction at the same operation as mastectomy. This bears on donor-site selection as well, since it is precisely in the older and less robust patient that the shorter, pedicled operation is easiest to justify.

### 4.3. Chest Wall Reconstruction as a Forced Operation

The fourteen coverage-intent patients require interpretation on their own terms. The defining feature of this group is the extent of resection. Removal of locally advanced or recurrent disease from the chest wall leaves a defect whose closure becomes the immediate surgical requirement, and the reconstructive plan follows the resection rather than shaping it. The surgeon selects a method capable of covering the defect reliably and quickly; the esthetic considerations that govern breast reconstruction fall away almost entirely. Free tissue transfer was for that reason not undertaken in any of these patients, and every defect was covered with a pedicled or local option.

The consequence for outcome interpretation is direct. Entry into this group is determined by tumor extent, so the group is defined by advanced disease before any reconstructive decision is made. The mortality of 28.6% therefore measures the oncological situation that made the operation necessary rather than any hazard conferred by the operation itself. Reporting these patients within a breast reconstruction series, or entering them into a comparison of reconstructive techniques, would attribute to a flap what properly belongs to the disease. Stage was unrecorded for eight of the fourteen patients, a pattern consistent with referral for salvage after treatment begun elsewhere or after recurrence remote from the original staging episode; this incompleteness reinforces rather than weakens the argument for separation, since it indicates that these patients enter the reconstructive service along a different clinical pathway. These fourteen patients constituted 0.6% of the cohort, 10.3% of its recorded mortality, and 8.1% of its breast-cancer-specific mortality. Pooling them into a breast reconstruction series inflates apparent mortality and, more insidiously, contaminates any comparison between reconstructive techniques, since the flaps used for chest wall coverage are the same flaps used for breast reconstruction. The distinction is one of indication rather than of tissue.

### 4.4. Prosthetic Reconstruction, Radiotherapy, and the Choice Between Single-Stage and Two-Stage Reconstruction

Two different measures of burden point in opposite directions in this series. Prosthetic reconstruction was offered to the most oncologically favorable patients and carried the lowest recorded mortality, at 0.4% after direct-to-implant reconstruction; the same technique nevertheless generated the heaviest reoperation burden, with 10.5% of patients returning to the operating room for a complication, compared with 1.4% after a mini latissimus dorsi flap and none after a lateral thoracodorsal flap. The presence of a prosthesis, rather than the magnitude of the reconstruction, governed the likelihood of further surgery. Counting operations rather than patients would obscure this contrast entirely, since it would merge a planned expander exchange with an unplanned washout for infection.

The radiotherapy data require careful reading, since two distinct questions are easily conflated. The first asks whether irradiating a prosthetic reconstruction increases the likelihood of further surgery. Our data answer affirmatively at the level of association: complication reoperation rose from 9.3% to 19.1% among implant recipients receiving post-reconstruction radiotherapy, while autologous reconstruction showed no comparable difference. The irradiated group was nonetheless a different population, with stage II or higher disease in 59.0% against 28.1% and neoadjuvant chemotherapy in 27.3% against 7.1%; radiation, extent of resection, and systemic treatment therefore remain entangled. Attributing the excess to radiation alone would overstate what these data support.

The second question is the one that actually confronts a surgeon planning an operation: given that PMRT is anticipated, should the prosthetic reconstruction be staged? This question can be addressed here only within the limits of a retrospective, single-center comparison resting on few events. Among irradiated patients, complication reoperation occurred after 18.0% of direct-to-implant reconstructions and 23.8% of two-stage expander reconstructions (*p* = 0.55), numerically favoring the single-stage approach; the two strategies were likewise indistinguishable among non-irradiated patients (9.2% vs. 7.9%) and across the whole prosthetic cohort (10.8% vs. 13.6%, *p* = 0.51). In this cohort, therefore, two-stage reconstruction did not demonstrate a measurable advantage over single-stage direct-to-implant reconstruction, including among patients receiving radiotherapy. That observation rests on five events among twenty-one irradiated expander reconstructions; it cannot, on that basis, carry recommendations about surgical planning, although it is consistent with accumulating evidence that single-stage reconstruction performs at least as well as two-stage reconstruction in irradiated patients [27,28]. The fixed-window analysis of Section 3.5, which restricts each comparison to patients with equal opportunity for observation, gave concordant results: reoperation for a complication remained more frequent after implant-based reconstruction than after autologous or oncoplastic reconstruction at every window, and the one-year comparison among irradiated prosthetic reconstructions (9.4% after direct-to-implant reconstruction, 5.0% after two-stage insertion) remained far from demonstrating any advantage for staging. Capsular contracture remains a recognized consequence of irradiating a prosthesis, yet its occurrence appears amenable to operative technique and falls short of placing direct-to-implant reconstruction beyond consideration. An anticipated requirement for PMRT was for many years treated as a reason in itself to avoid immediate definitive prosthetic reconstruction, either by placing a tissue expander or by preferring autologous reconstruction outright. That convention has been changing, and the expander pathway carries a burden of its own: expander-related infection, exposure, deflation, and delayed exchange are all well described, and the additional operation both adds risk and postpones adjuvant treatment in a proportion of patients.

Practice at this institution has moved in that direction, and the movement bears on the figures reported here. Expander insertion was proportionally commoner in the earlier years, when anticipated irradiation drove the choice; the later years are dominated by immediate direct-to-implant reconstruction, performed in patients some of whom subsequently required irradiation. Two-stage reconstruction in this series is now largely confined to the delayed setting. The comparison between the two prosthetic strategies should therefore be read as a comparison between two strategies applied in different eras as well as to different patients. A residual group remains for whom radiotherapy is not anticipated at mastectomy and becomes necessary only when the pathological stage exceeds what the preoperative assessment predicted; for these patients the sequence cannot be planned in advance, and the observation that single-stage reconstruction was not measurably disadvantaged after irradiation in this cohort is of practical interest, while requiring confirmation in larger series.

The combined latissimus dorsi and implant group requires separate comment, since it carried the highest complication reoperation rate in the series (17.5%). Three features bear on that figure. The technique places a prosthesis beneath a flap and therefore carries both the device-related reoperation indications of implant-based reconstruction and the donor-site morbidity of a flap; seroma was the most commonly recorded complication. It was also irradiated after reconstruction more often than direct-to-implant reconstruction (28.1% vs. 15.9%). Selection into the technique was moreover not neutral: it was used where flap volume alone was judged insufficient and, in a small number of patients, to restore volume while covering a defect after infection or skin-flap necrosis had compromised an earlier reconstruction. The confidence interval around the point estimate is correspondingly wide (9.8–29.4), and the figure should be read as consistent with, rather than exceeding, the burden carried by prosthetic reconstruction generally.

A caution attaches to the category of implant-based reconstruction as used here, since grouping every prosthetic reconstruction together conceals variation that governs outcome. The implant may sit prepectorally or in a subpectoral pocket, the subpectoral pocket may be created with partial or with complete inferior release of the pectoralis major, and radiotherapy may or may not include a sequential chest wall boost. Each of these is a plausible determinant of capsular contracture and of implant-threatening complications, and none was captured reliably in this dataset: the plane was specified in only 177 of 633 implant-based procedures, and no record distinguished a sequential boost. The association reported here between irradiation and reoperation should therefore be read as a category-level average taken across configurations of differing risk. The practical implication is that the decision governing outcome may lie in pocket configuration and in the delivery of a boost rather than in the choice of a prosthesis or of a single-stage approach in itself, and that this question requires a dedicated analysis with the relevant variables recorded prospectively.

### 4.5. Lapsed Insurance Eligibility as a Surrogate for Mortality

Our fourth finding is methodological. Termination of National Health Insurance eligibility is sometimes treated in Korean clinical practice as an informal indication that a patient has died, on the reasonable ground that mortality does terminate eligibility. Our data indicate that the inference fails in reverse. The eligibility-terminated group proved indistinguishable from survivors on stage, neoadjuvant treatment, reconstructive strategy, and age, and differed sharply from the confirmed deceased on all of these. Had we classified these 41 patients as deceased, the apparent mortality of the cohort would have more than doubled and the stage gradient would have been diluted, with the observed mortality of stage 0/I disease rising from 0.6% to 2.6%.

Four considerations support holding the two groups apart. The two annotations are generated by different processes: vital status was entered by the treating team from institutional records, whereas eligibility is an administrative field that changes for emigration, prolonged residence abroad, transfer to the Medical Aid program, and loss of nationality, as well as for death, and nothing in the record identifies which applies to an individual patient. The empirical profile of the eligibility-terminated group moreover does not behave as a group of unrecognized deaths should, since a group composed largely of deaths would be expected to carry the disease burden of the deceased. The group also clusters in the earlier years of the series, the pattern expected of an administrative field that accumulates lapses with elapsed time rather than of an outcome that tracks disease. The attendance record supplies the fourth and most direct consideration. Every one of the 41 patients attended this institution in person after the index reconstruction, 15 of them during the final two and a half years of the series and five during 2026, and more than a third of those final attendances were at departments with no part in breast cancer follow-up, among them obstetrics and gynecology, psychiatry, and endocrinology. That is the ordinary attrition of a follow-up clinic rather than the signature of unrecognized death. The conclusion is not that all 41 patients are known to be alive today, since attendance establishes survival only as far as the date recorded, and the date of the lapse itself was unavailable; the conclusion is that the group as a whole does not behave as a group of deaths. Korean administrative data do contain a reliable mortality signal: the National Health Insurance Service eligibility database carries a date of death, and resident registration expiration data from the Ministry of the Interior and Safety agree with national vital statistics to within 0.4% [29,30]. Studies requiring mortality endpoints should link to one of these sources rather than infer mortality from the absence of insurance coverage.

### 4.6. Limitations

The most important limitation concerns the denominator rather than the numerator. Dates of the terminal event were available for every deceased patient, and a date of last attendance was retrieved for the 41 patients whose insurance eligibility had terminated, yet dates of last contact for the remaining survivors were absent, so person-time could not be computed, and formal survival analysis remained impossible. Mortality appears as a status recorded at any point during a follow-up period varying from a few weeks to thirteen years, and patients operated on recently have had little opportunity to be observed to die; the 2024–2026 cohorts contribute 21.3% of survivors and only 2.6% of the recorded mortality events. The absolute mortality figures should therefore be read as a lower bound on cumulative mortality rather than as a survival estimate [31]. Ascertainment also depended on institutional records and is likely to be incomplete, since patients who die elsewhere may escape capture. Staging data were missing for 24.2% of patients, and the multivariable model rested on 29 events, so the odds ratios are imprecise and should be regarded as exploratory. Radiotherapy and chemotherapy were recorded as binary variables without dose, fractionation, target volume, or agent; no record distinguished a sequential chest wall boost, and the reconstructive plane was specified in only 177 of 633 implant-based procedures; these variables therefore function as prognostic markers rather than as estimable treatment effects. The classification was derived from free-text operative descriptions and may misclassify individual operations. Finally, this is a single-institution series from two surgeons with a strong preference for pedicled thoracic flaps, and its findings may not generalize directly to other institutions or populations. The technique mix reflects the small-to-moderate breast volumes prevailing in Korean women [15,16], an institutional depth of experience with pedicled thoracic donor sites, and a reimbursement environment in which the National Health Insurance Service has covered post-mastectomy reconstruction and the associated acellular dermal matrix since 2015 [2,3]; centers in which abdominally based free-flap reconstruction predominates, as in many Western series, or in which coverage rules differ, should expect a different spectrum, although the direction of change after reimbursement matches Korean claims-based analyses [2,3]. Complications short of reoperation, recurrence, and endocrine or targeted therapy escaped systematic capture, as did the cause-level detail of complication reoperations (infection, exposure, capsular contracture, rupture, or asymmetry), which the free-text record did not support reliably. Patient-reported outcomes deserve explicit mention: breast reconstruction is ultimately judged by quality of life, body image, symmetry, and satisfaction, and no validated instrument such as the BREAST-Q was administered in this cohort, so mortality and reoperation capture only part of what these operations are performed to achieve.

One period of the series requires separate comment. From 20 February 2024, the great majority of residents in Korean training hospitals resigned in protest at a government plan to expand medical school admissions, and normal staffing was not restored until the second half of 2025. Tertiary centers curtailed elective operations in response, and our caseload reflects it: index reconstructive procedures fell from 185 in 2023 to 121 in 2024 before recovering to 174 in 2025 and 160 over the first seven months of 2026. The contraction is unlikely to have fallen evenly across techniques, since a long autologous reconstruction is harder to sustain without resident assistance than a shorter prosthetic or oncoplastic procedure. Part of the apparent acceleration away from lengthy flap surgery in the final era may therefore reflect this staffing constraint rather than reconstructive preference alone, and the 2024 and 2025 figures should be interpreted with that in mind.

## 5. Conclusions

Over thirteen years, reconstructive breast surgery at this center required twenty-six distinct techniques, shifted decisively from autologous to implant-based reconstruction, and sustained a large oncoplastic workload that national mastectomy-indexed statistics fail to capture. The five latissimus dorsi designs served clearly separable indications and warrant separate reporting. Recorded mortality varied widely across techniques; the gradient tracked disease burden rather than the operation and is best understood as confounding by indication. The same caution applies to the treatment variables: the higher mortality recorded after neoadjuvant chemotherapy and after irradiation preceding reconstruction reflects the indications for those treatments and must not be read as evidence of harm. Chest wall reconstruction emerged as a small, distinct, and prognostically severe category that merits separate reporting from breast reconstruction. Lapse of National Health Insurance eligibility proved a poor surrogate for mortality and requires replacement by linkage to a validated mortality source.

## Figures and Tables

**Figure 1:**
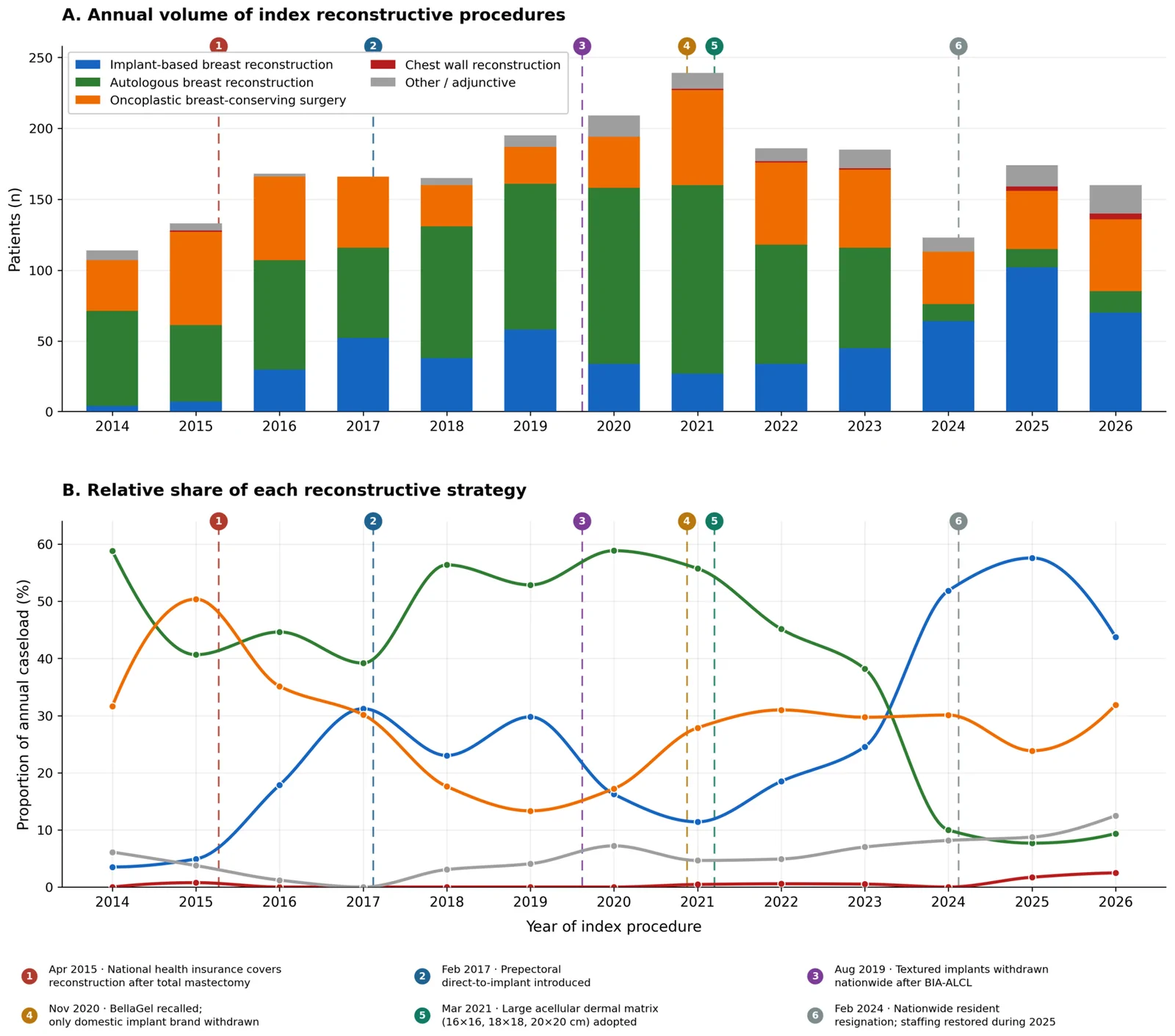
Evolution of reconstructive practice, 2014–2026. (**A**) Annual number of index procedures by reconstructive strategy. The index procedure is the first reconstructive operation recorded for each patient, so the annual totals include coverage and adjunctive procedures as well as the three restorative strategies. (**B**) The same data are shown as a proportion of each year’s caseload. The 2012 and 2013 cases (n = 2) are omitted for scale, and data for 2026 cover January to July only. Six events that changed the range of available options, or the capacity to deliver them, are marked on both panels and are discussed in Section 4.2: (1) April 2015, reimbursement of post-mastectomy reconstruction and of acellular dermal matrix; (2) February 2017, first prepectoral direct-to-implant reconstruction at this institution; (3) August 2019, nationwide withdrawal of textured implants; (4) November 2020, recall of BellaGel; (5) March 2021, adoption of large-format acellular dermal matrix; (6) February 2024, start of the nationwide resident resignation. Of these, events (1), (3), and (4) are the three change points specified in advance for the segmented regression described in Section 2.7. In both panels, colors denote the reconstructive strategies shown in the legend of panel (A), and the numbered dashed vertical lines mark the six events listed above.

**Figure 2:**
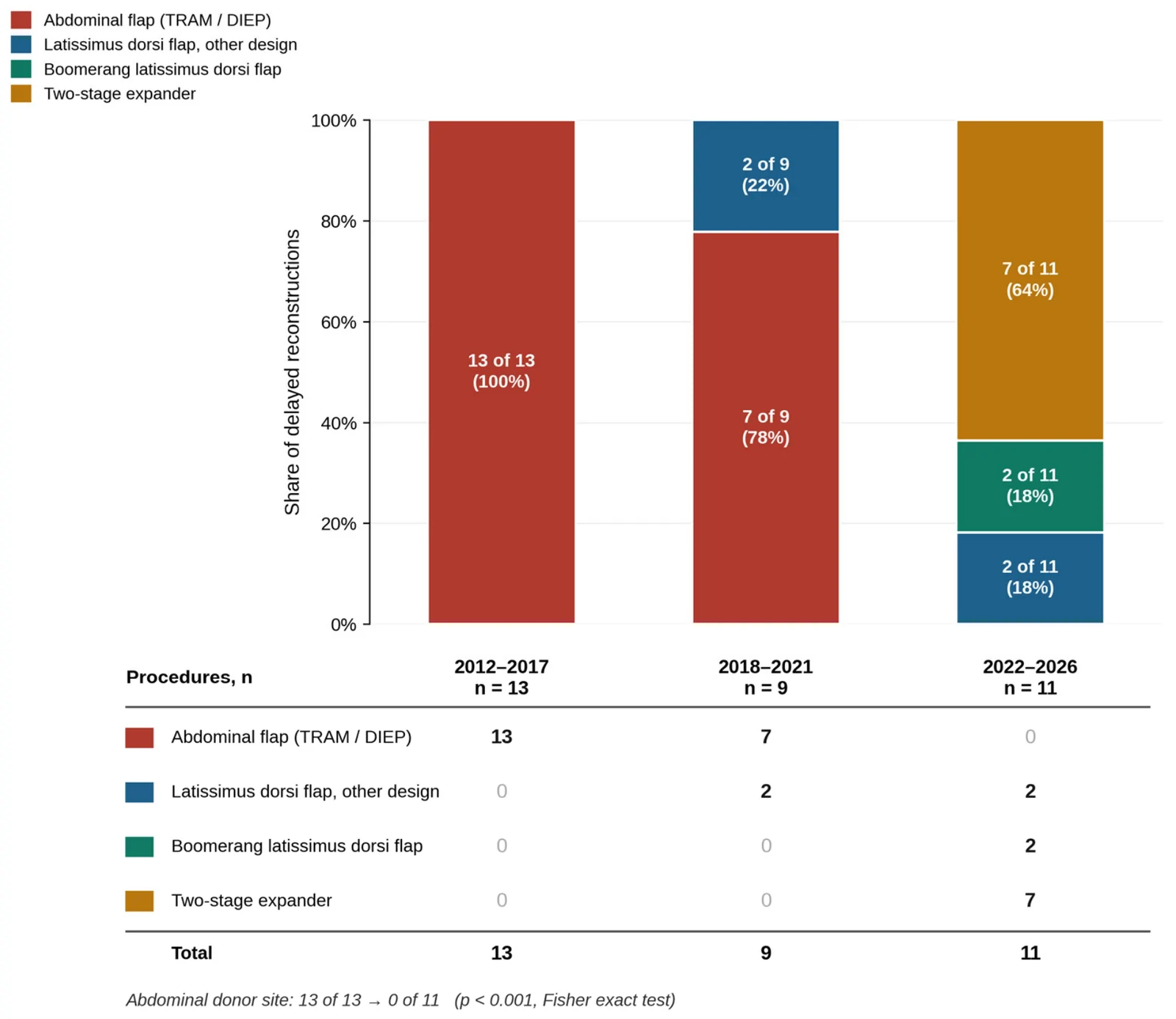
Donor site used for delayed breast reconstruction, by era. Bars show the share of delayed reconstructions accounted for by each donor site; the counts on which those shares are based are given beneath. Denominators are small, so the counts rather than the percentages should be read as the primary quantity. Cases were identified by explicit annotation of the operation as delayed in the operative record and are therefore a lower bound on all non-immediate reconstruction. No delayed reconstruction was recorded in 2020 or 2024. DIEP, deep inferior epigastric perforator; TRAM, transverse rectus abdominis myocutaneous.

**Figure 3:**
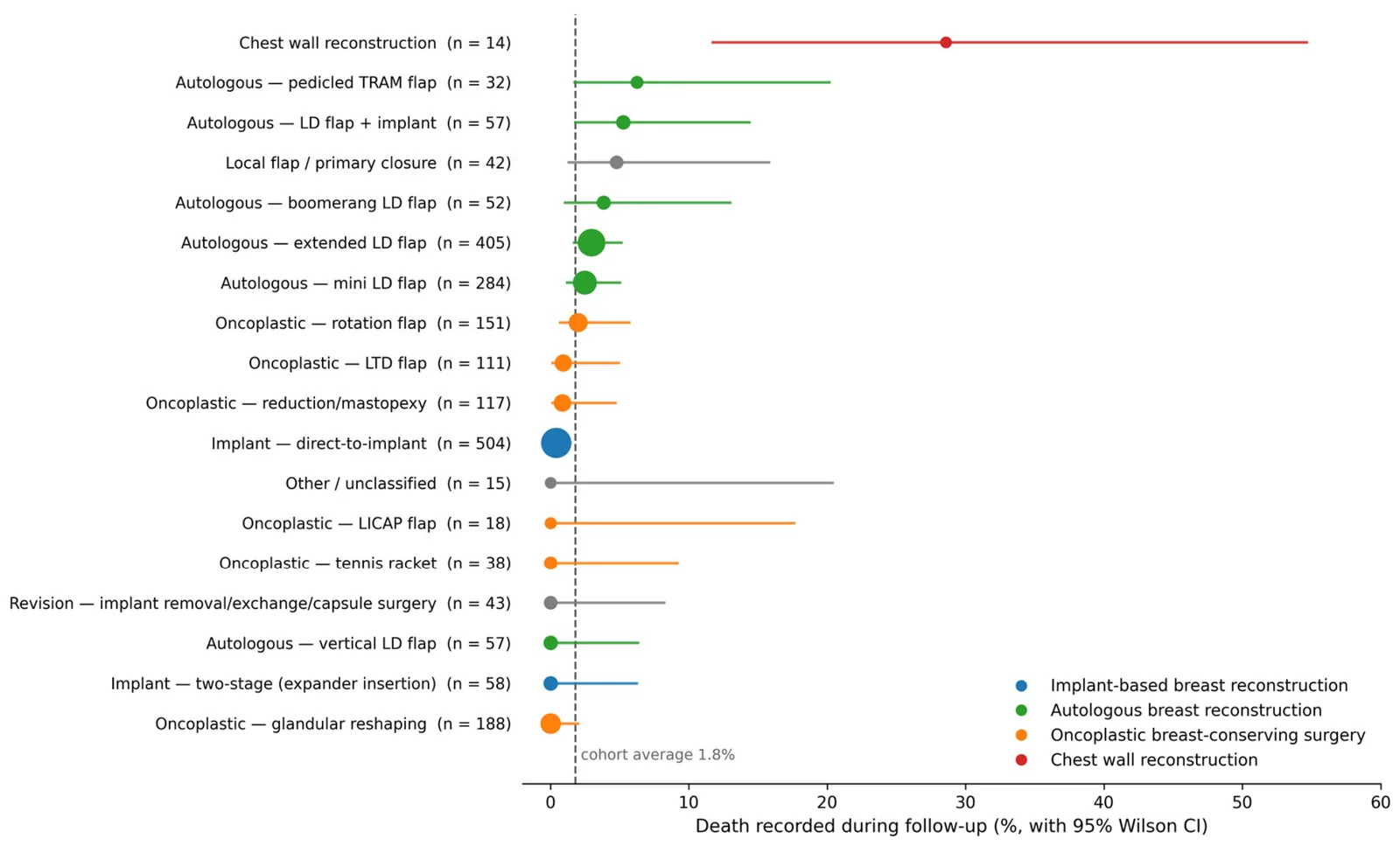
Recorded mortality by primary reconstructive technique among techniques performed in at least 14 patients. Points are proportions with Wilson 95% confidence intervals; point area is proportional to the number of patients and color denotes reconstructive strategy, with grey denoting other procedures (local flap/primary closure, implant revision/capsule surgery, and unclassified procedures). The dashed line marks the cohort mortality of 1.8%. The patient counts underlying each point, and the proportions shown, are the patient-level figures of Table 2 and Section 3.8.

**Figure 4:**
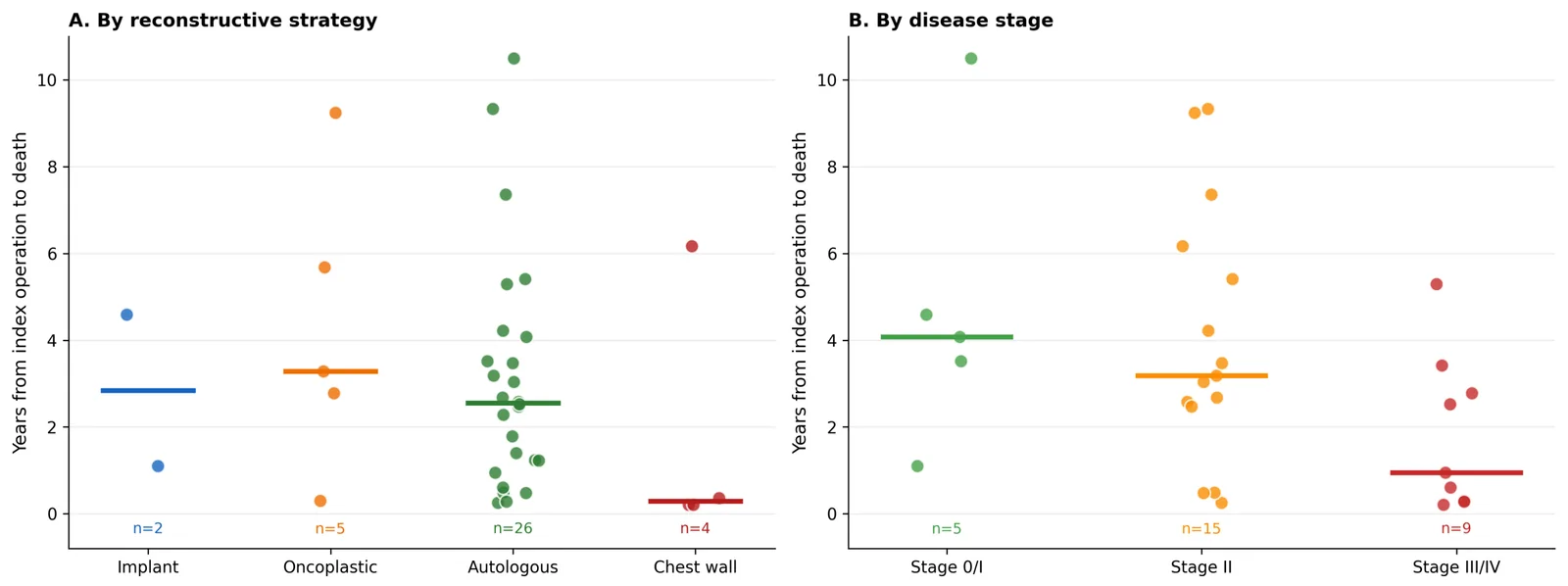
Interval from the index reconstruction to the recorded mortality event among the 39 deceased patients, by reconstructive strategy (**A**) and by disease stage (**B**). Each point is one patient, and the horizontal bar is the group median. These are intervals among patients known to be deceased rather than survival estimates, since dates of last contact for surviving patients were unavailable.

**Figure 5:**
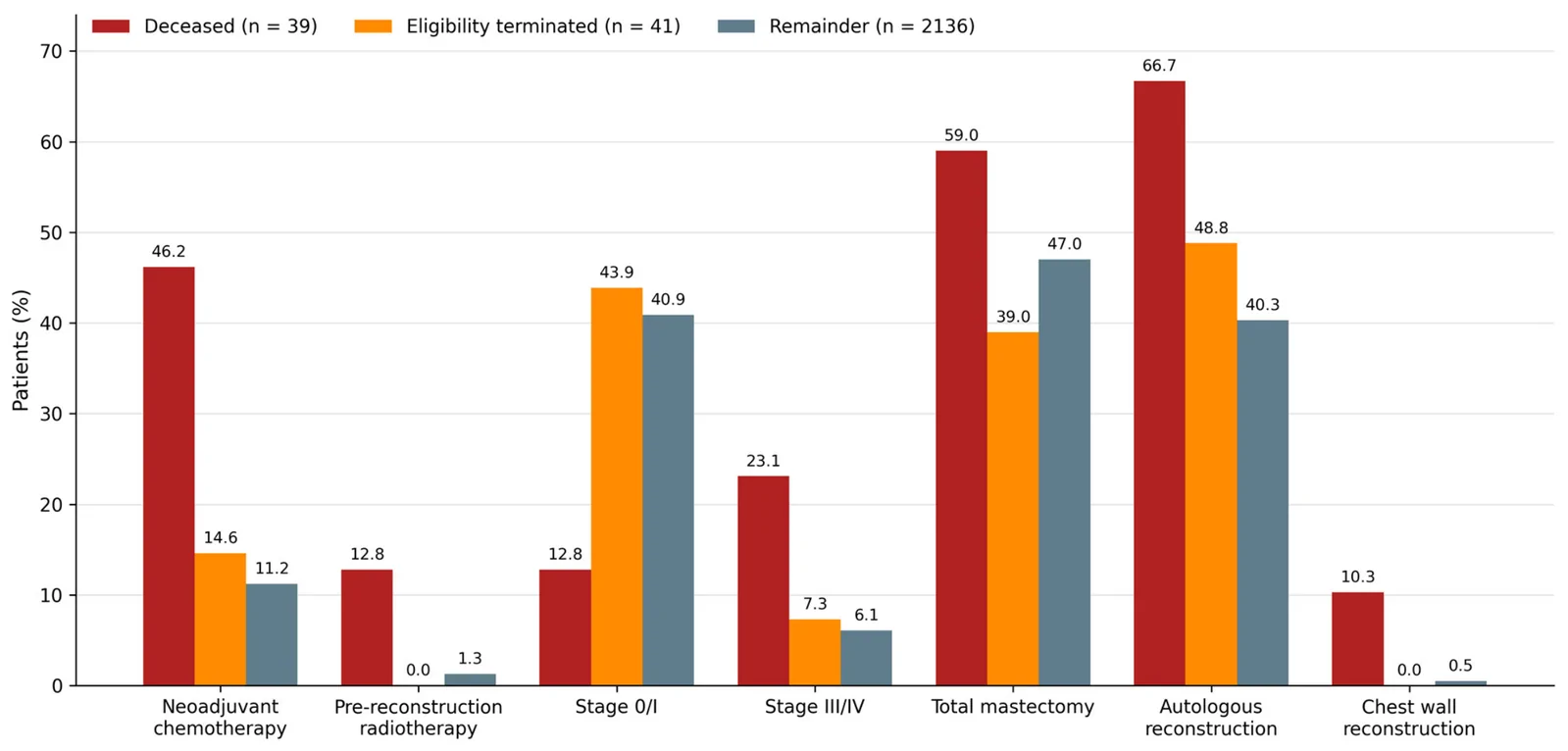
Disease-burden profile of the three vital-status groups. Bars show the proportion of individuals carrying the specified characteristic within each group: the confirmed deceased (n = 39), the patients whose National Health Insurance eligibility had terminated (n = 41), and the remainder of the cohort (n = 2136). The corresponding numerical values and *p*-values are given in Table 9.

**Table 1:** Baseline characteristics of the cohort (2216 patients; 2523 procedures).

Characteristic	n/Value	%
**Demographics (per patient)**		
Age, years, mean ± SD (median, IQR)	47.6 ± 8.5 (47, 42–52)	Range: 14–85
Height, cm, mean ± SD	159.1 ± 5.5	
Weight, kg, mean ± SD	58.6 ± 9.4	
Body mass index, kg/m^2^, mean ± SD	23.1 ± 3.4	
Body mass index ≥ 25 kg/m^2^	536	24.2
Breast volume, mL, median (IQR)	290 (220–400)	4092 breasts in 2075 patients
**Oncological surgery**		
Total mastectomy	1042	47.0
Partial mastectomy	1029	46.4
Bilateral mixed	9	0.4
Not recorded	136	6.1
**Histology**		
Invasive ductal carcinoma	1293	58.3
Ductal carcinoma in situ	514	23.2
Invasive lobular carcinoma	73	3.3
Other/not specified	336	15.2
**Stage**		
0/I	897	40.5
II	639	28.8
III/IV	143	6.5
Not recorded	537	24.2
**Systemic and radiation therapy**		
Neoadjuvant chemotherapy	263	11.9
Palliative preoperative chemotherapy (M1)	12	0.5
Adjuvant chemotherapy	843	38.0
No chemotherapy	868	39.2
Pre-reconstruction radiotherapy	32	1.4
Post-reconstruction radiotherapy	967	43.6
No irradiation	976	44.0
**Vital status recorded**		
Mortality recorded	39	1.8
Insurance eligibility terminated	41	1.9

IQR, interquartile range; SD, standard deviation. Percentages use the full cohort of 2216 patients as the denominator. Missing values appear explicitly rather than being excluded. Breast volume is reported per breast rather than per patient, since the two breasts of a patient were measured separately; measurements were made by water displacement from an adhesive-tape cast. The youngest patient in the series was a 14-year-old girl who underwent partial mastectomy for a fibroadenoma with immediate oncoplastic glandular reshaping; the waiver of consent granted by the Institutional Review Board covered all patients irrespective of age at operation.

**Table 2:** The reconstructive technique spectrum, with the patient and disease profile of each technique.

Technique	Procedures, n	Patients, n	Age (Years)	BMI (kg/m^2^)	TM (%)	Stage ≥ II (%)	Neoadj. CTx (%)	Post-Recon. RTx (%)	Deceased, n (%)
**Restorative intent—implant-based**									
Direct-to-implant	529	504	48.1 ± 8.3	22.3 ± 3.0	98.3	33.9	8.5	15.9	2 (0.4)
Two-stage expander insertion	59	58	49.2 ± 8.8	22.6 ± 2.9	92.7	63.9	20.7	36.2	0 (0.0)
Two-stage expander exchange	45	2	—	—	—	—	—	—	—
**Restorative intent—autologous, latissimus dorsi**									
Extended latissimus dorsi flap	419	405	45.9 ± 7.4	22.8 ± 3.1	87.0	50.7	21.0	34.3	12 (3.0)
Mini latissimus dorsi flap	287	284	46.1 ± 6.7	22.9 ± 2.9	1.4	49.6	11.3	68.3	7 (2.5)
Latissimus dorsi flap + implant	64	57	46.4 ± 7.9	23.7 ± 3.7	98.2	44.9	7.0	28.1	3 (5.3)
Vertical latissimus dorsi flap	58	57	48.0 ± 8.6	23.2 ± 3.2	0.0	48.8	8.8	68.4	0 (0.0)
Boomerang latissimus dorsi flap	55	52	48.8 ± 7.9	23.9 ± 3.6	90.0	53.8	7.7	19.2	2 (3.8)
Latissimus dorsi flap, design not specified	4	1	—	—	—	—	—	—	—
**Restorative intent—autologous, abdominal and perforator**									
Pedicled TRAM flap	33	32	46.0 ± 6.8	24.6 ± 3.4	100.0	42.9	34.4	31.2	2 (6.2)
Free TRAM flap (muscle-sparing)	12	12	—	—	—	—	—	—	—
DIEP flap	5	4	—	—	—	—	—	—	—
TDAP flap	3	3	—	—	—	—	—	—	—
**Restorative intent—oncoplastic, volume displacement**									
Glandular reshaping	235	188	48.1 ± 9.3	23.4 ± 3.8	1.1	43.7	5.3	61.2	0 (0.0)
Rotation flap	156	151	50.0 ± 8.6	23.6 ± 3.3	2.7	57.6	9.3	64.2	3 (2.0)
Reduction mammoplasty/mastopexy	151	117	46.7 ± 9.6	26.2 ± 4.5	4.7	51.1	12.8	57.3	1 (0.9)
Tennis-racket method	39	38	46.3 ± 8.6	23.1 ± 3.4	0.0	38.9	2.6	86.8	0 (0.0)
**Restorative intent—oncoplastic, volume replacement**									
Lateral thoracodorsal flap	114	111	48.5 ± 7.6	23.4 ± 2.9	0.9	46.9	10.8	71.2	1 (0.9)
LICAP flap	18	18	45.4 ± 6.6	23.9 ± 4.1	0.0	52.9	11.1	55.6	0 (0.0)
**Coverage intent**									
Chest wall reconstruction	14	14	61.6 ± 11.3	23.9 ± 1.7	90.9	100.0	42.9	28.6	4 (28.6)
**Adjunctive**									
Revision—implant/capsule surgery	186	43	55.9 ± 9.5	22.0 ± 2.6	87.5	66.7	0.0	2.3	0 (0.0)
Contralateral balancing	146	—	—	—	—	—	—	—	—
Local flap/primary closure	92	42	48.7 ± 14.2	22.7 ± 3.7	27.8	43.8	9.5	31.0	2 (4.8)
Nipple–areola reconstruction	83	4	—	—	—	—	—	—	—
Revision—wound or scar	50	2	—	—	—	—	—	—	—
Fat grafting/liposuction	25	2	—	—	—	—	—	—	—
Other/unclassified	18	15	40.1 ± 13.1	22.5 ± 3.8	42.9	40.0	6.7	26.7	0 (0.0)
Total	2900	2216							

BMI, body mass index; CTx, chemotherapy; DIEP, deep inferior epigastric perforator; LICAP, lateral intercostal artery perforator; Neoadj., neoadjuvant; Post-recon. RTx, post-reconstruction radiotherapy; TDAP, thoracodorsal artery perforator; TM, total mastectomy; TRAM, transverse rectus abdominis myocutaneous. Techniques are grouped by reconstructive intent and strategy. Procedure counts exceed the number of operations because a single operation may combine techniques, and a patient may undergo more than one technique in separate operations or in a second reconstruction episode. The primary technique is assigned from the index operation using a fixed clinical hierarchy, so the two count columns differ; the strategy totals used elsewhere (implant-based 564, autologous 907, oncoplastic 623, chest wall reconstruction 14) count each patient once. The patient and disease profile is shown for techniques serving as the primary technique in at least 14 patients; a dash denotes a technique below that threshold, or one that is almost never a patient’s primary technique. Percentages for total mastectomy and stage use patients with that variable recorded as denominator. The LICAP flap is a perforator flap but is classified as oncoplastic volume replacement throughout, since it was used to replace volume after breast-conserving surgery rather than to reconstruct the breast after mastectomy.

**Table 3:** Patient and disease characteristics by reconstructive strategy.

Strategy	n	Age (Years)	BMI (kg/m^2^)	TM (%)	Stage ≥ II (%)	Neoadj. CTx (%)	Post-Recon. RTx (%)	Deceased, n (%)
**Restorative intent**	**2094**							
Implant-based	564	48.2 ± 8.4	22.4 ± 3.0	97.9	36.5	9.8	17.9	2 (0.4)
Autologous	907	46.4 ± 7.3	23.1 ± 3.2	54.9	50.1	15.9	45.6	26 (2.9)
Oncoplastic	623	48.2 ± 8.9	24.0 ± 3.8	2.0	49.1	8.7	64.4	5 (0.8)
**Coverage intent**	**14**							
Chest wall reconstruction	14	61.6 ± 11.3	23.9 ± 1.7	90.9	100.0	42.9	28.6	4 (28.6)
**Adjunctive**	**108**							
Other/adjunctive	108	50.7 ± 12.5	22.5 ± 3.3	39.6	52.8	4.5	18.9	2 (1.8)

BMI, body mass index; CTx, chemotherapy; Post-recon. RTx, post-reconstruction radiotherapy. Age compared by Kruskal–Wallis test; categorical variables by chi-square test. Stage and total mastectomy percentages use patients with that variable recorded as denominator; for chest wall reconstruction, the extent of resection was recorded in 11 of the 14 patients and the stage was recorded in 6.

**Table 4:** Reoperation burden by primary reconstructive technique.

Primary Technique	Patients	Operations	Operations Per Patient	Any Reoperation, n (%)	Reoperation for Complication, n (%)	95% CI
Direct-to-implant	504	616	1.22	87 (17.3)	53 (10.5)	8.1–13.5
Extended latissimus dorsi flap	405	450	1.11	40 (9.9)	14 (3.5)	2.1–5.7
Mini latissimus dorsi flap	284	298	1.05	10 (3.5)	4 (1.4)	0.5–3.6
Glandular reshaping	188	197	1.05	9 (4.8)	5 (2.7)	1.1–6.1
Rotation flap	151	156	1.03	4 (2.6)	1 (0.7)	0.1–3.7
Reduction mammoplasty/mastopexy	117	118	1.01	1 (0.9)	0 (0.0)	0.0–3.2
Lateral thoracodorsal flap	111	112	1.01	1 (0.9)	0 (0.0)	0.0–3.3
Two-stage expander insertion	58	129	2.22	49 (84.5)	8 (13.8)	7.2–24.9
Latissimus dorsi flap + implant	57	75	1.32	17 (29.8)	10 (17.5)	9.8–29.4
Vertical latissimus dorsi flap	57	58	1.02	1 (1.8)	0 (0.0)	0.0–6.3
Boomerang latissimus dorsi flap	52	59	1.13	4 (7.7)	3 (5.8)	2.0–15.6
Revision—implant/capsule surgery	43	47	1.09	2 (4.7)	1 (2.3)	0.4–12.1
Local flap/primary closure	42	42	1.00	0 (0.0)	0 (0.0)	0.0–8.4
Tennis-racket method	38	40	1.05	2 (5.3)	0 (0.0)	0.0–9.2
Whole cohort	2216	2523	1.14	240 (10.8)	101 (4.6)	3.8–5.5

CI, confidence interval. The 95% CI is the Wilson interval for the reoperation-for-complication proportion in the preceding column. Techniques performed in at least 38 patients as the primary technique are shown, a higher threshold than that used in Table 2 because a reoperation proportion estimated in fewer patients carries an uninformatively wide confidence interval. Operations per patient counts every operation recorded for that patient, including staged completions and adjunctive procedures. Any reoperation denotes the proportion of patients returning to the operating room for any indication; reoperation for complication denotes the proportion undergoing at least one operation in which a complication formed the indication or one component of it, planned staged completions and nipple–areola reconstructions having been excluded. This patient-level count therefore exceeds the number of operations whose principal indication was a complication (Section 3.5).

**Table 5:** Post-reconstruction radiotherapy, reconstructive strategy, and reoperation for complication.

Comparison	Post-Reconstruction RTx	No Irradiation	*p*-Value
**Reoperation for complication, by post-reconstruction radiotherapy status**			
Implant-based reconstruction	21/110 (19.1%)	42/451 (9.3%)	0.006
Direct-to-implant reconstruction	16/89 (18.0%)	38/411 (9.2%)	0.023
Autologous reconstruction	11/427 (2.6%)	17/480 (3.5%)	0.45
**Disease burden of implant recipients**			
Stage II or higher	59.0%	28.1%	
Neoadjuvant chemotherapy	27.3%	7.1%	

All comparisons used the Fisher exact test. Figures for reoperation are the number and proportion of patients undergoing at least one subsequent operation for a complication; planned staged completions and nipple–areola reconstructions are excluded. Denominators are restricted to patients whose radiotherapy status was recorded and are therefore smaller than the group sizes given in Table 2 and Table 3. For direct-to-implant reconstruction, radiotherapy status was recorded in 500 of the 504 patients of Table 2 and Table 4, and for two-stage expander insertion in 59 of 61 expander reconstruction units (the 58 patients of Table 2 and Table 4, together with three expander units in patients whose primary technique was classified elsewhere); the denominators of this table and of Section 3.6 are these restricted counts. Panel B compares the disease burden of implant recipients who did and did not receive post-reconstruction radiotherapy, and shows that the two columns describe different populations rather than a randomized contrast. The unit in this table is the reconstruction rather than the patient, since radiotherapy was recorded in relation to a given reconstruction; the counts are therefore not directly comparable with the patient-level counts in Table 4.

**Table 6:** The fourteen patients who underwent chest wall reconstruction.

Case	Age (Years)	Stage	Neoadjuvant CTx	Vital Status	Coverage Technique
1	57	NR	Yes	Deceased	Bilateral advancement flap
2	74	IIA	No	Deceased	Bilateral advancement flap
3	40	NR	Yes	Alive	Local flap with split-thickness skin graft
4	49	NR	Yes	Deceased	Thoracoabdominal flap
5	62	IIIA	No	Alive	Extended latissimus dorsi flap
6	58	IIIC	Yes	Alive	Vertical latissimus dorsi flap
7	67	NR	NR	Alive	Boomerang latissimus dorsi flap
8	68	IV	No	Alive	Rotation flap
9	46	IIA	Yes	Alive	Primary closure
10	64	IV	Yes	Deceased	Mini boomerang latissimus dorsi flap
11	67	NR	NR	Alive	Extended latissimus dorsi flap
12	72	NR	NR	Alive	Bilateral advancement flap
13	81	NR	NR	Alive	Bilateral advancement flap
14	58	NR	NR	Alive	Extended latissimus dorsi flap

CTx, chemotherapy; NR, not recorded. Stage was recorded in six of the fourteen patients. The extent of the oncological resection is not tabulated, since the partial-versus-total mastectomy classification used elsewhere does not discriminate among these patients; the single case coded as partial mastectomy in the source dataset was recorded by the oncological team as a mastectomy. Coverage technique is transcribed from the operative record; cases 1 and 2 were recorded only as “chest wall reconstruction” and were established on individual review as bilateral advancement flaps, and case 7 was recorded as a latissimus dorsi musculocutaneous flap and confirmed on the same review as a boomerang latissimus dorsi flap raised for chest wall coverage. Case 5 was recorded by the oncological team as wide excision with chest wall resection, and case 1 followed modified radical mastectomy. One further operation recorded as chest wall reconstruction is not included here, since it was performed together with an implant exchange in a patient whose index reconstruction had been implant-based; that patient is counted under implant-based reconstruction throughout. Three of the four terminal events followed progression of breast cancer and occurred 75, 77, and 132 days after coverage; case 2 had an unrelated metastatic pancreatic carcinoma 2252 days after coverage, so breast-cancer-specific mortality in this group was 3 of 14 (21.4%).

**Table 7:** Recorded mortality: univariable comparison and multivariable logistic regression.

Variable	Deceased (n = 39)	Others (n = 2177)	*p*
**Univariable comparison**			
Age, years, mean ± SD	46.1 ± 9.6	47.6 ± 8.6	0.25
Body mass index, kg/m^2^	24.4 ± 4.0	23.1 ± 3.4	0.051
**Stage**			**<0.001**
0/I	5 (0.6% of stratum)	892	
II	15 (2.3%)	624	
III/IV	9 (6.3%)	134	
Neoadjuvant chemotherapy	18 (46.2%)	245 (11.3%)	<0.001
Palliative preoperative chemotherapy	2 (5.1%)	10 (0.5%)	0.008
Pre-reconstruction radiotherapy	5 (12.8%)	27 (1.2%)	<0.001
Adjuvant chemotherapy	14 (35.9%)	829 (38.1%)	0.92
Post-reconstruction radiotherapy	19 (48.7%)	948 (43.5%)	0.62
Total mastectomy	23 (59.0%)	1019 (46.8%)	0.056
Operating surgeon (A vs. B)	15/9	843/749	0.47
**Reconstructive strategy**			**<0.001**
Implant-based	2 (0.4% of stratum)	562	
Oncoplastic	5 (0.8%)	618	
Autologous	26 (2.9%)	881	
Chest wall reconstruction	4 (28.6%)	10	
Other/adjunctive	2 (1.8%)	106	
**Multivariable logistic regression**			
Covariate	Odds ratio	95% CI	*p*
**Model 1—with neoadjuvant chemotherapy**			
Age, per year	0.97	0.92–1.02	0.233
Stage II (ref. 0/I)	3.56	1.28–9.96	0.015
Stage III/IV (ref. 0/I)	5.25	1.53–18.07	0.008
Neoadjuvant chemotherapy	2.34	0.99–5.50	0.051
Implant-based (ref. autologous)	0.22	0.05–0.97	0.046
Oncoplastic (ref. autologous)	0.37	0.12–1.12	0.078
Chest wall reconstruction (ref. autologous)	14.43	1.84–113.00	0.011
**Model 2—without neoadjuvant chemotherapy**			
Age, per year	0.96	0.91–1.01	0.111
Stage II (ref. 0/I)	3.96	1.43–10.95	0.008
Stage III/IV (ref. 0/I)	7.35	2.26–23.87	0.001
Implant-based (ref. autologous)	0.22	0.05–0.94	0.041
Oncoplastic (ref. autologous)	0.34	0.11–1.01	0.052
Chest wall reconstruction (ref. autologous)	16.11	2.12–122.63	0.007

SD, standard deviation. Continuous variables were compared with the Mann–Whitney U test and categorical variables with the chi-square test. In the univariable comparison: percentages in the deceased column refer to that column unless marked “of stratum”, in which case they express mortality within the stratum. Chemotherapy and radiotherapy are mutually exclusive timing categories, so a patient treated both before and after operation is counted only in the preoperative row. In the multivariable regression: CI, confidence interval; OR, odds ratio. Both models were fitted in 1636 patients with complete data on all covariates, containing 29 events. Model 1 is the primary model and retains neoadjuvant chemotherapy, defined as preoperative chemotherapy in a patient without M1 disease. Model 2 omits neoadjuvant chemotherapy. Given the low event count, the estimates from both models are exploratory. Adjuvant therapies showed no association with mortality on univariable analysis and were therefore omitted.

**Table 8:** Cause of the terminal event in the 39 deceased patients.

Cause of the Terminal Event	Patients, n	% of the Deceased (n = 39)	Reconstructive Strategy
Breast cancer progression	37	94.9	—
Metastatic breast carcinoma	35	89.7	Implant 2, oncoplastic 5, autologous 23, chest wall 3, other 2
Breast carcinoma with a concurrent second primary	2	5.1	Autologous 2
Malignancy other than breast carcinoma	2	5.1	—
Metastatic malignant phyllodes tumor	1	2.6	Autologous 1
Metastatic pancreatic carcinoma	1	2.6	Chest wall 1

The two patients with a concurrent second primary had documented progression of both malignancies, one of breast carcinoma with metastatic squamous cell carcinoma of the pelvic nodes and one of breast carcinoma with cervical carcinoma; both are counted as breast-cancer-related. The patient with a malignant phyllodes tumor had a breast malignancy that is not a carcinoma, and the patient with pancreatic carcinoma had a wholly unrelated malignancy six years after chest wall reconstruction; both are excluded from the breast-cancer-specific analyses.

**Table 9:** Testing lapsed insurance eligibility as a surrogate for mortality.

Variable	Deceased (n = 39)	Eligibility Terminated (n = 41)	Remainder (n = 2136) *	*p*
Neoadjuvant chemotherapy	46.2%	14.6%	11.2%	0.0031
Pre-reconstruction radiotherapy	12.8%	0.0%	1.3%	0.024
Stage 0/I	12.8%	43.9%	40.9%	0.0028
Stage III/IV	23.1%	7.3%	6.1%	0.063
Total mastectomy	59.0%	39.0%	47.0%	0.12
Autologous reconstruction	66.7%	48.8%	40.3%	0.12
Chest wall reconstruction	10.3%	0.0%	0.5%	0.052
Age, years, mean	46.1	48.0	47.6	0.31
**Index operation before 2021**	**64.1%**	**87.8%**	**51.0%**	**—**

* *p*-values compare the deceased with the eligibility-terminated group (Fisher exact test, or Mann–Whitney for age). Comparisons of the eligibility-terminated group with the remainder reached significance for none of the variables examined. Neoadjuvant chemotherapy is defined as in Table 7, that is, excluding the twelve patients with M1 disease whose preoperative chemotherapy was classified as palliative.

**Table 10:** Last recorded attendance at this institution in the 41 patients whose National Health Insurance eligibility had terminated.

Characteristic	Patients, n	%
Interval from index reconstruction, years, median (IQR)	4.3 (1.8–7.5)	Range: 0.4–10.6
**Setting of the last attendance**		
Outpatient clinic	35	85.4
Inpatient admission	4	9.8
Emergency department	2	4.9
**Calendar year of the last attendance**		
2017–2020	12	29.3
2021–2023	14	34.1
2024	5	12.2
2025	5	12.2
2026	5	12.2
**Department of the last attendance**		
Medical oncology	13	31.7
Breast and thyroid surgery	7	17.1
Plastic and reconstructive surgery	5	12.2
Obstetrics and gynecology	4	9.8
Endocrinology	2	4.9
Gastroenterology	2	4.9
Psychiatry	2	4.9
Emergency medicine	2	4.9
Family medicine	1	2.4
Neurosurgery	1	2.4
Neurology	1	2.4
Nuclear medicine	1	2.4
**Other**		
Transferred to another institution at the last attendance	3	7.3

IQR, interquartile range. Percentages use the 41 eligibility-terminated patients as the denominator. Attendance in person establishes that the patient was alive on the date shown; the date on which insurance eligibility lapsed was not held in the record, so attendance and lapse could not be ordered.

## Data Availability

The data presented in this study are available on reasonable request from the corresponding author. The data are not publicly available because they contain information that could compromise the privacy of the individuals who participated in the study and because their release is restricted by institutional policy.

## Supplementary Materials

The following supporting information can be downloaded at: https://www.mdpi.com/article/10.3390/medicina62101871/s1, Table S1: The 26 technique categories, their definitions, and the assignment rules; Table S2: Segmented logistic regression of reconstructive practice over time; Figure S1: Share of index reconstructions accounted for by implant-based and autologous reconstruction.

## Abbreviations

The following abbreviations are used in this manuscript:

ADM	acellular dermal matrix
BIA-ALCL	breast implant–associated anaplastic large cell lymphoma
BMI	body mass index
CI	confidence interval
DIEP	deep inferior epigastric perforator
DTI	direct-to-implant
IQR	interquartile range
IRB	institutional review board
LD	latissimus dorsi
LICAP	lateral intercostal artery perforator
LTD	lateral thoracodorsal
NHIS	National Health Insurance Service
OR	odds ratio
PMRT	post-mastectomy radiotherapy
SD	standard deviation
TDAP	thoracodorsal artery perforator
TRAM	transverse rectus abdominis myocutaneous
