# Supplementary material for: Thirteen Years of Reconstructive Breast Surgery in a Korean Tertiary Practice: Procedure Spectrum, Shifting Practice, and Modality Selection in 2216 Patients
Source: Medicina (Kaunas). 2026 Sep 27;62(10):1871. doi: 10.3390/medicina62101871 (PMC13706941; doi:10.3390/medicina62101871)
Supplement: Supplementary file 1 [file medicina-62-01871-s001.zip › medicina-4534011-supplementary.pdf]

Supplementary Materials

Thirteen Years of Reconstructive Breast Surgery in a Korean Tertiary Practice: Procedure Spectrum, Shifting Practice, and Modality Selection in 2216 Patients

Table S1. The 26 technique categories, their definitions, and the assignment rules.

Tier	Category	Definition	Assignment rule (normalized operative text)
Restorative—implant-based	Direct-to-implant	A permanent silicone implant placed at the same operation as the mastectomy, prepectorally or in a subpectoral pocket.	"silicone implant insertion", "implant insertion", "breast reconstruction with silicone implant"; plane qualifiers (prepectoral, subpectoral, anterior sling) retained but not used for assignment.
	Two-stage expander insertion	A tissue expander placed at the time of mastectomy as the first stage of a planned two-stage reconstruction.	"tissue expander insertion", "expander insertion".
	Two-stage expander exchange	Removal of an expander and insertion of the permanent implant as the planned second stage.	"tissue expander removal" together with "implant insertion".
Restorative—autologous	Extended latissimus dorsi flap	Latissimus dorsi muscle harvested with the surrounding fatty envelope to reconstruct a whole breast after total mastectomy.	"extended LD flap"; also endoscopic or robotic maximal harvest.
	Mini latissimus dorsi flap	A limited muscle segment used to fill a partial defect after breast-conserving surgery, entered through a separate axillary incision.	"mini LD flap", "robot assisted mini LD flap", "endoscopic assisted mini LD flap".
	Vertical latissimus dorsi flap	Extended harvest with a vertically oriented skin paddle.	"vertical LD flap", "vertical mini LD flap".
	Boomerang latissimus dorsi flap	Institutional modification reorienting the skin paddle to increase transferable skin area and projection.	"boomerang LD flap", "mini boomerang LD flap".
	Latissimus dorsi flap + implant	A latissimus dorsi flap of any design with a silicone implant placed beneath it at the same operation.	Any latissimus dorsi term co-occurring with an implant term in the same operative record.
	Latissimus dorsi flap, design not specified	A latissimus dorsi flap recorded without a named design.	"LD flap" or "latissimus dorsi flap" with no design qualifier.
	Pedicled TRAM flap	Transverse rectus abdominis myocutaneous flap transferred on its superior epigastric pedicle.	"pedicled TRAM", "delayed pedicled TRAM".
	Free TRAM flap (muscle-sparing)	Muscle-sparing free transverse rectus abdominis myocutaneous transfer.	"free TRAM", "muscle sparing free TRAM".
	DIEP flap	Deep inferior epigastric perforator flap.	"DIEP".
	TDAP flap	Thoracodorsal artery perforator flap with the muscle preserved.	"TDAP".
Restorative—oncoplastic	Glandular reshaping	Volume displacement by mobilization and advancement of glandular flaps to obliterate the lumpectomy cavity.	"glandular reshaping", "oncoplastic breast surgery with glandular reshaping".
	Rotation flap	Rotation of a de-epithelialized parenchymal flap into the defect.	"rotation flap", "subcutaneous rotation flap".

Tier	Category	Definition	Assignment rule (normalized operative text)
	Reduction mammoplasty/mastopexy	Therapeutic reduction or mastopexy in which the tumor is resected within a reduction pattern.	"reduction mammoplasty", "mastopexy" without the qualifier "contralateral".
	Lateral thoracodorsal flap	Volume replacement by a fasciocutaneous flap from the lateral chest wall.	"LTD flap", "lateral thoracodorsal flap".
	Tennis-racket method	Circumareolar incision with a radial extension over the tumor, permitting resection and closure through one incision.	"tennis racket method".
	LICAP flap	Lateral intercostal artery perforator flap with the muscle preserved.	"LICAP".
Coverage	Chest wall reconstruction	Coverage of a soft-tissue defect that resisted primary closure after wide resection, the object being durable closure rather than breast form.	"chest wall reconstruction" in the operative record, confirmed individually; one case recorded only as a latissimus dorsi musculocutaneous flap was assigned on review.
Adjunctive	Revision—implant/capsule surgery	Removal, exchange, capsulotomy, or capsulectomy performed on an existing prosthesis.	"implant removal", "implant change", "capsulectomy", "capsulotomy" without a concurrent reconstructive term.
	Contralateral balancing	Reduction, mastopexy, or augmentation of the unaffected breast for symmetry.	"contralateral" with reduction, mastopexy, or augmentation.
	Nipple–areola reconstruction	Local C-V flap, free nipple graft, or areolar tattooing.	"nipple reconstruction", "C-V flap", "areolar tattooing".
	Local flap/primary closure	Direct closure or a small local flap where no reconstructive intent beyond wound closure was recorded.	"primary closure", "local flap", "excision and primary closure", "advancement flap" without oncoplastic qualifier.
	Revision—wound or scar	Revision of a wound, scar, or inframammary fold.	"wound revision", "scar revision", "IMF revision".
	Fat grafting/liposuction	Correction of contour irregularity by lipofilling, with or without liposuction.	"fat injection", "liposuction".
Residual	Other/unclassified	Operative text that could not be assigned to any category above; 18 of 2900 technique components (0.6%).	No matching pattern.

Operative descriptions were lower-cased and normalized for recurrent misspellings ("recsontruction", "impalnt", "wih") before matching. Where an operation contained elements of more than one category, a fixed clinical hierarchy assigned the reconstructive intent of highest complexity as the primary technique: coverage intent, then autologous, then oncoplastic, then implant-based, then adjunctive. A single operation may contribute to more than one technique count at procedure level; each patient receives exactly one primary technique from the index operation.

Table S2. Segmented logistic regression of reconstructive practice over time.

Term	Odds ratio per year	95% CI	p
Implant-based reconstruction			
Slope, Dec 2012 – Mar 2015	2.69	0.84–8.60	0.094
Slope, Apr 2015 – Aug 2019	1.41	1.24–1.60	<0.001
Slope, Sep 2019 – Oct 2020	0.28	0.20–0.39	<0.001

Term	Odds ratio per year	95% CI	p
Slope, Nov 2020 – Jul 2026	1.58	1.46–1.70	<0.001
Change in slope at Apr 2015 (NHIS coverage)	0.52	0.16–1.76	0.296
Change in slope at Aug 2019 (textured implants withdrawn)	0.20	0.13–0.30	<0.001
Change in slope at Nov 2020 (BellaGel recall)	5.73	3.86–8.51	<0.001
Autologous reconstruction			
Slope, Dec 2012 – Mar 2015	0.43	0.26–0.73	0.002
Slope, Apr 2015 – Aug 2019	1.17	1.06–1.28	0.002
Slope, Sep 2019 – Oct 2020	1.41	1.06–1.88	0.019
Slope, Nov 2020 – Jul 2026	0.57	0.52–0.61	<0.001
Change in slope at Apr 2015 (NHIS coverage)	2.69	1.52–4.77	0.001
Change in slope at Aug 2019 (textured implants withdrawn)	1.21	0.85–1.72	0.296
Change in slope at Nov 2020 (BellaGel recall)	0.40	0.29–0.56	<0.001

The unit is the index reconstruction of each of the 2216 patients and time runs in years from 22 December 2012. Slopes are odds ratios per year within the segment; change-in-slope terms are the ratio by which the annual odds ratio is multiplied after the change point. The three change points were specified in advance from documented external events and were not selected from the data. A specification adding a level shift at each change point improved fit (likelihood-ratio p = 0.001) without altering the direction or the ordering of the slope changes. The series is uncontrolled and single-center, so these estimates identify coincidence in time rather than cause.

Figure S1. Share of index reconstructions accounted for by implant-based and autologous reconstruction.

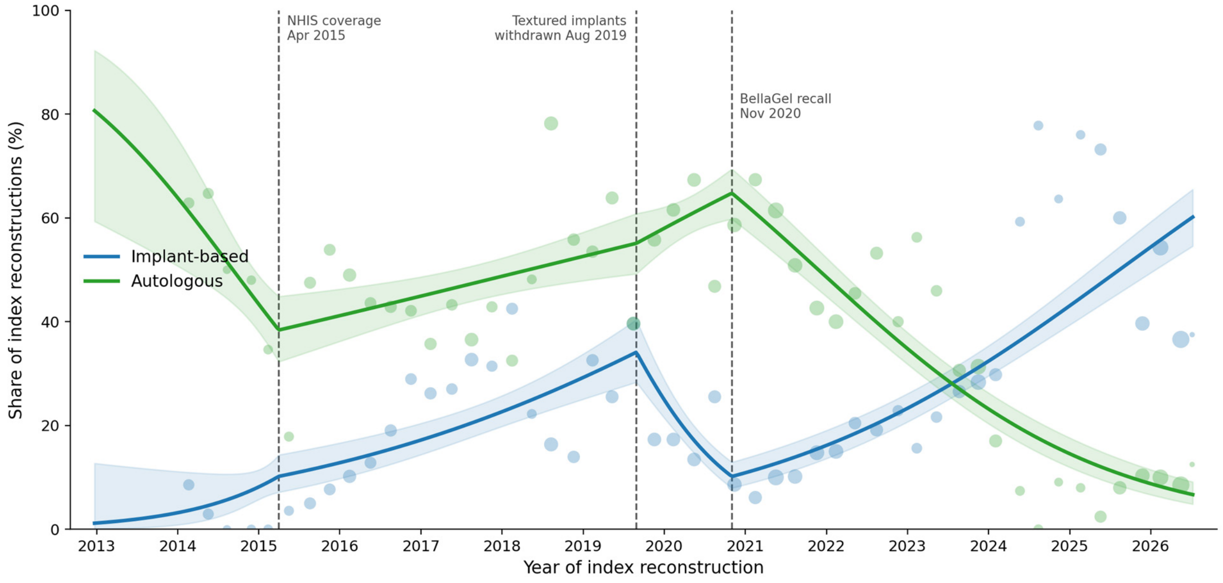

Circles are observed quarterly proportions, sized by the number of index reconstructions in the quarter; quarters with fewer than five reconstructions are omitted. Lines are fitted values from the segmented logistic model with 95% confidence bands. Dashed verticals mark the three change points specified in advance.
